# Development and Validation for Quantification of 7-Nitroso Impurity in Sitagliptin by Ultraperformance Liquid Chromatography with Triple Quadrupole Mass Spectrometry

**DOI:** 10.3390/molecules27238581

**Published:** 2022-12-05

**Authors:** Hari Naga Prasada Reddy Chittireddy, J. V. Shanmukha Kumar, Anuradha Bhimireddy, Mohammed Rafi Shaik, Merajuddin Khan, Syed Farooq Adil, Mujeeb Khan, Fatimah N. Aldhuwayhi

**Affiliations:** 1 Department of Engineering Chemistry, College of Engineering, Koneru Lakshmaiah Education Foundation, Vaddeswaram, Guntur 522502, Andhra Pradesh, India; 2 Aurobindo Pharma Limited, Sanga Reddy, Indrakaran 502329, Telangana, India; 3 Department of Chemistry, College of Science, King Saud University, P.O. Box 2455, Riyadh 11451, Saudi Arabia

**Keywords:** sitagliptin, 7-nitroso impurity, nitrosamine, UHPLC-MS/MS, ICH

## Abstract

The purpose of this research study was to develop an analytical method for the quantification of 7-nitroso-3-(trifluoromethyl)-5,6,7,8-tetrahydro-[1,2,4] triazolo [4,3-a] pyrazine (7-nitroso impurity), which is a potential genotoxic impurity. Since sitagliptin is an anti-diabetic medication used to treat type 2 diabetes and the duration of the treatment is long-term, the content of nitroso impurity must be controlled by using suitable techniques. To quantify this impurity, a highly sensitive and reproducible ultraperformance liquid chromatography with triple quadrupole mass spectrometry (UHPLC-MS/MS) method was developed. The analysis was performed on a Kromasil-100, with a C18 column (100 mm × 4.6 mm with a particle size of 3.5 µm) at an oven temperature of approximately 40 °C. The mobile phase was composed of 0.12% formic acid in water, with methanol as mobile phases A and B, and the flow rate was set to 0.6 mL/min. The method was validated according to the current International Council for Harmonisation (ICH) guidelines with respect to acceptable limits, specificity, reproducibility, accuracy, linearity, precision, ruggedness and robustness. This method is useful for the detection of the impurity at the lowest limit of detection (LOD), which was 0.002 ppm, and the lowest limit of quantification (LOQ), which was 0.005 ppm. This method was linear in the range of 0.005 to 0.06 ppm and the square of the correlation coefficient (R^2^) was determined to be > 0.99. This method could help to determine the impurity in the regular analysis of sitagliptin drug substances and drug products.

## 1. Introduction

In the process of producing pharmaceutical products, there is a high risk of product contamination from the starting materials, reaction byproducts and other impurities, which negatively influences the safety and toxicological profile of the final drug [1]. Additionally, the final products (active pharmaceutical ingredients) may also produce genotoxic impurities due to degradation during synthesis, the formulation of dosage forms, storage, and aging, etc. [2]. For example, the generation of contaminants may be due to the degradation of penicillins and cephalosporins, etc. [3]. Among various impurities, organic contaminants that can potentially induce genetic mutations, chromosomal breaks, etc., are regarded as potential genotoxic impurities, which can be responsible for causing cancers in humans [4]. Therefore, the generation of these impurities must be controlled and monitored, which is typically attempted during the process of production [5]. However, the complete removal of these contaminants is often not assured, and thus the process must be carefully monitored to avoid unnecessary clinical holds or delays caused by regulatory agencies [6]. Therefore, the development of analytical techniques for the accurate analysis and detection of genotoxic impurities in pharmaceuticals is both imperative and challenging for analysts and scientists [7].

The defined limits for genotoxic impurities are recommended in the ICH Q3A guidelines [8]. These genotoxic impurities include nitrosamines. The ICH M7 (R1) [9] guideline defines N-nitrosamines as substances in the “cohort of concern”, which are medicinal products that have limits referred to as the substance-specific acceptable intake (AI) (the threshold of toxicological concern (TTC) has a value of 1.5 ug/day and this cannot be applied). Therefore, it is recommended that such nitroso-compounds should be controlled as they are mutagenic carcinogen impurities.

**Figure 1:**
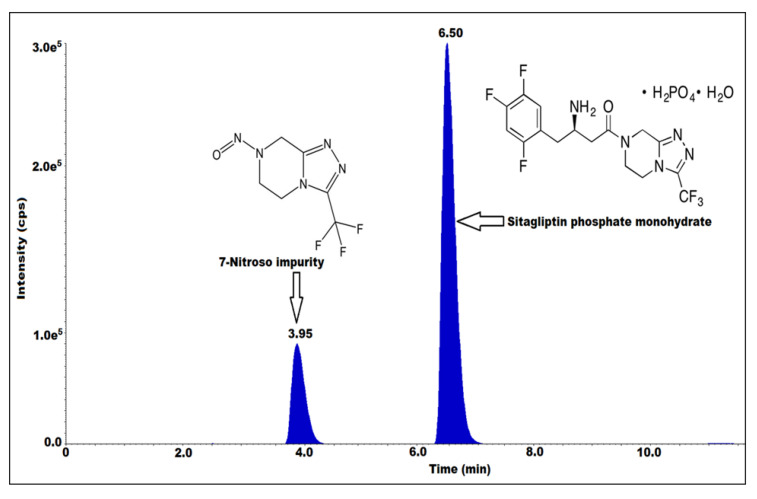
Structures of sitagliptin phosphate monohydrate and 7-nitroso impurity with MRM spectrum.

Sitagliptin phosphate monohydrate (Figure 1) is chemically known as 7-[(3R)-3-amino-1-oxo-4-(2,4,5-trifluorophenyl)butyl]-5,6,7,8-tetrahydro-3-(trifluoromethyl)-1,2,4-triazolo [4,3-a] pyrazine phosphate (1:1) monohydrate [10]. It is an anti-diabetic drug used in the treatment of type 2 diabetes and it belongs in the class of dipeptidyl peptidase-4 (DPP-4) inhibitors. It works by increasing insulin production and decreasing the production of glucagon in the pancreas. The U.S. Food and Drug Administration (FDA) [11] approved sitagliptin in October 2006 [12]. In the United States of America, Merck & Co market the drug under the name of JANUVIA.

In sitagliptin, product-related nitrosamine impurities are present in the form of 7-nitroso-3-(trifluoromethyl)-5,6,7,8-tetrahydro-[1,2,4]triazolo[4,3-a] pyrazine (Figure 1). This research study is significant because if a drug product contains even a single N-nitrosamine, which is a reported 7-nitroso impurity and a potential genotoxic impurity (according to the EMA) with a limit of 37 ng/day [13], it can have a harmful long-term impact on humans.

All of the regulatory agencies, such as FDA, EMA and other agencies, were directed to control N-nitroso impurities and the FDA has recalled several products since 2018 including ARBs, ranitidine, nizatidine, and metformin. Therefore, very sensitive analytical approaches are required to detect and identify very low levels of these impurities. In the literature, various methods are reported for the analysis of sitagliptin drug substances and drug products including the SFC-MS/MS [14], RP-HPLC [15,16], HPTLC [17], HPLC [18,19,20,21], HPLC-CD [22], LC-UV, LC-MS, and FT-IR method [23], as well as the UV spectrophotometric [24] and UPLC-MS/MS [25] method; however, these are not sophisticated enough to analyze a particular 7-nitroso impurity. A review of the literature revealed that there is no specific method available for the analysis of 7-nitroso impurity in sitagliptin. Therefore, we developed an analytical method that is precise and simple, which was also validated for the determination of this impurity.

In order to develop the method, we trialed various analytical techniques such as the HPLC and GC-MS/MS methods to identify and quantify the impurity at the ppm level; however, these methods were found to be unacceptable as the results were poor or there was no response. The analytical techniques used in the HPLC method included photodiode-array detection (PDA), phenyl, C8_,_ and C18 columns, which were used to quantify the impurity. Furthermore, the development trials were performed with buffers with different pH (acidic, basic, and neutral) and solvents. In this method, the impurity concentration was observed to be more than 150 ppm, with the lowest response in the sample concentration. Based on the results, we concluded that the HPLC method was not suitable for the quantification of this impurity.

With regard to the GC-MS/MS method, mass tuning was performed by using an EI ion source. Very poor fragmentation and ionization were observed. The development trials were performed by using a scan method using USP phase G43, a mid-polar 6% cyanopropyl, 94% polydimethylsiloxane, with a column of 60 m × 0.32 mm × 1.8 µm (length, inner diameter and film thickness). However, no peak response was observed. Based on the results, we concluded that the GC-MS/MS method was not suitable for the quantification of this impurity. The UHPLC-MS/MS method presented here is a new, advanced and industrially feasible method for the identification and quantification of the impurity.

## 2. Results and Discussions

### 2.1. Optimization of Mass Spectrometric Parameters

**Figure 2:**
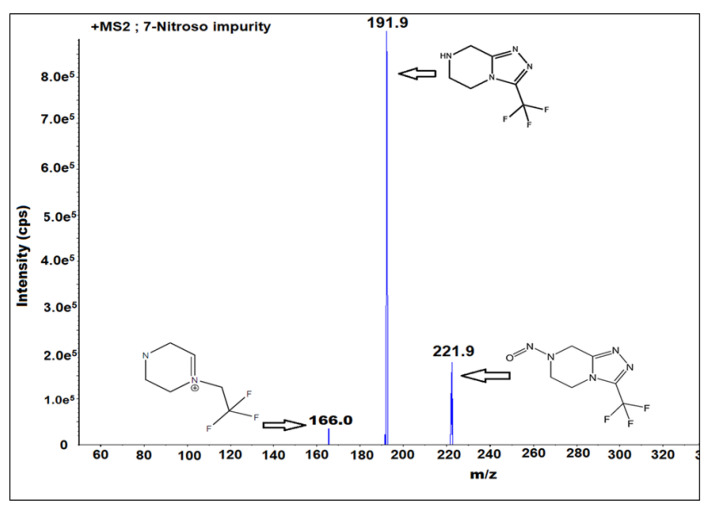
Fragmentation pattern of 7-nitroso impurity m/z 221.9, N-NO bond cleavage and formation of m/z 191.9 and probability of nitrogen molecule elimination from m/z 191.9 and formation of m/z 166.0 in the mass ionization chamber.

Optimization of the mass parameters played a critical role in the method development. The interpretation and selection of mass fragments played a key role in the identification and analysis at the sub ppm (parts per million) and ppb (parts per billion) level of impurity analysis. Mass tuning was performed for sitagliptin and 7-nitroso impurities to identify the Q1 and Q3 values. It was performed by using different ion sources such as positive atmospheric pressure chemical ionization (APCI), negative APCI, positive ESI, and negative ESI. The Q1 value is 221.9 and Q3 value is 191.9 for 7-nitroso impurity for the MRM mode with an ESI ion source and positive ion polarity. The other mass parameters are DP 40, EP 10, CE 15, CAD medium, GS1 is the nebulizer gas 45 and the MS temperature is 400 °C. The solubility of the analytes was checked by using mass compatible solvents such as water, methanol, and acetonitrile. Sitagliptin and 7-nitroso impurities are soluble in water. The mass fragmentation pattern (Figure 2) of the impurity was identified.

### 2.2. Optimization of Chromatographic Conditions

The chromatographic conditions were established by using different mass compatible solvents and buffers. Different volatile acidic and basic buffers were used. For example, we used ammonia and formic acid in combination with different solvents such as methanol and acetonitrile as the mobile phase, different HPLC column chemistries (C8, C18, phenyl) and different column lengths (250, 150, 100 and 50 mm) and different particle size (5, 3.5 µm). Finally, the method was optimized by using 0.12% formic acid in water and methanol as mobile phase-A and mobile phase-B, with a gradient program and flow rate of 0.6 mL/min by using Kromasil-100, C18 with a particle size of 3.5 µm and an LC column with a length of 100 mm and diameter of 4.6 mm, the temperature was about 40 °C. The 7-nitroso impurity response and ionizations were very good under the above chromatographic and mass conditions, and the retention time was found to be about four minutes. To prevent mass source contamination from the peak concentration of sitagliptin, a diversion program of flow from the mass detector was applied after the elution of the 7-nitroso impurity peak (Table 1).

### 2.3. Method Validation Study

The final method was validated according to the ICH guidelines [26]. The validation parameters were system suitability and specificity, LOD, LOQ, LOQ precision, linearity, method precision, intermediate precision, accuracy, robustness and solution stability.

### 2.4. Specificity and System Suitability

**Figure 3:**
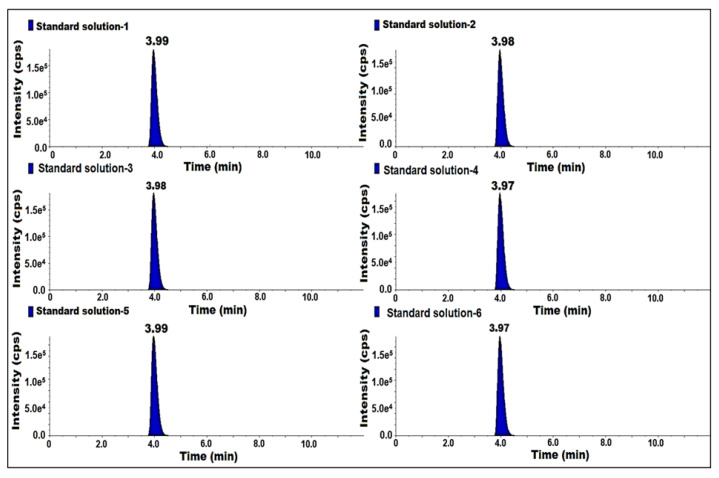
MS/MS MRM chromatogram of the system suitability standard.

As part of the validation process, the specificity and the system stability were assessed by injecting blank, sample 50 mg/mL, spiked sample, standard and individual impurity prepared at the specification level (0.03 ppm) into the diluent. The peak area percentage relative standard deviation (% RSD) of the standard was within the limit, no interference was observed at the retention time (RT) of 7-nitroso impurity in the blank. The retention time of the impurity in the sample, spiked sample, standard and individual impurity was about 4 min. Therefore, this method is specific (Table 2) (Figure 3).

### 2.5. LOD, LOQ and LOQ Precision

**Figure 4:**
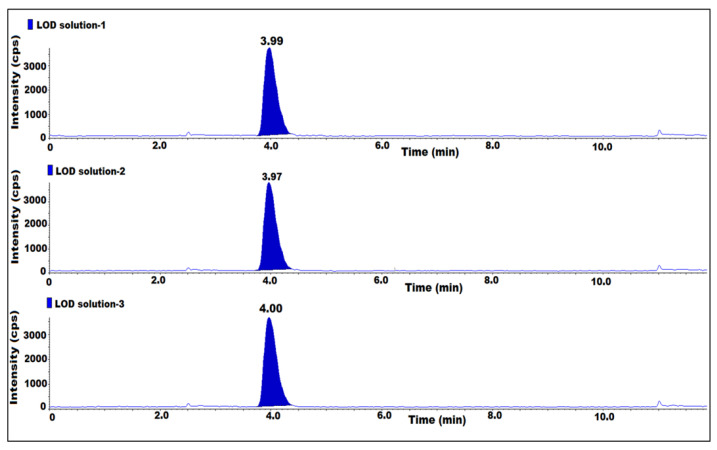
MS/MS MRM chromatogram of LOD solution.

The LOD and LOQ were established by injecting 7-nitroso impurity diluted solutions while taking the known concentration of the impurity, in triplicate. The final concentration of the LOD and LOQ with respect to the sample concentration was 0.002 ppm and 0.005 ppm, respectively, the signal-to-noise ratio (s/n) was equal to or greater than 3 for the LOD solutions and was equal to or greater than 10 for the LOQ solutions. The LOQ precision was assessed by injecting six replicate injections of LOQ solution. Based on the results, the s/n ratio was greater than 3 for the LOD and 10 for the LOQ solutions. The area %RSD for six replicate injections for LOQ precision was 3.1 (Table 2) (Figure 4).

### 2.6. Linearity and Range

**Figure 5:**
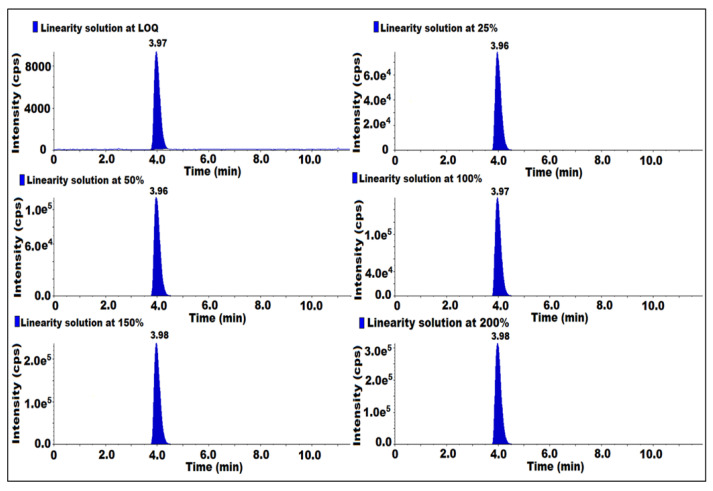
MS/MS MRM chromatogram of linearity solutions.

The linearity was established from LOQ to 200% of the concentration of 7-nitroso impurity with respect to sample concentration. LOQ was injected at 25, 50, 100, 150, 200% of six different known concentrations in duplicate. The linearity graph peak responses were plotted against the peak concentration of 7-nitroso impurity and the square of the correlation coefficient (r^2^) was evaluated. The obtained (r^2^) was 0.99. Hence, the method was proven to be linear (Table 2) (Figure 5).

### 2.7. Method Precision

The method precision (MP) was established by using a sitagliptin sample. Six samples were prepared at 50 mg/mL and six samples were prepared by spiking 7-nitroso impurity at the specification level, and all the solutions were injected. For each preparation, one injection was given. We determined the reproducibility of the results in regard to the presence of impurities in the samples, the spiked samples’ impurity content and the %RSD for the content of 7-nitroso impurity.

**Figure 6:**
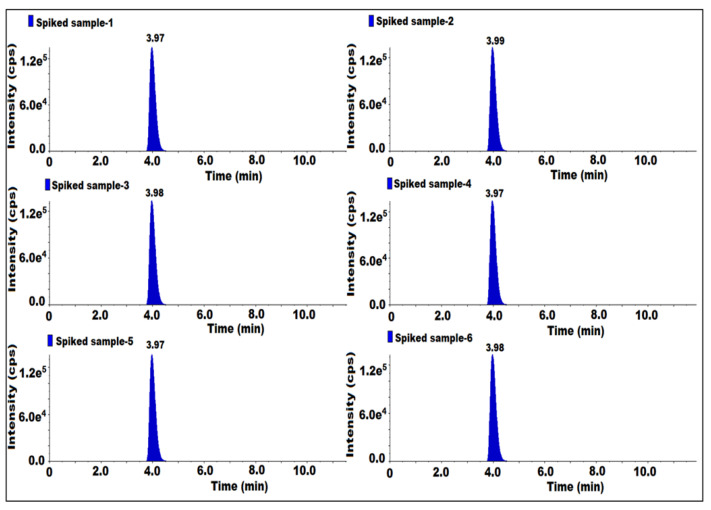
MS/MS MRM chromatogram of method precision.

As such, the samples did not have impurity and the results for the spiked impurity content are repeatable. The obtained content %RSD of spiked solution was 2.8 (Table 3). Hence, this method was found to be precise and repeatable (Figure 6).

### 2.8. Intermediate Precision

The intermediate precision (IP) was established by repeating the MP conditions with a different analyst, different day, different columns and preparations. The content and %RSD of the impurity was determined in sample and spike solutions. The spiked sample solutions (*n* = 6) %RSD was 3.2. The RSD (%) for preparations (*n* = 12) of MP and IP spiked sample at specification level was less than 20.0. The results indicated that the method was rugged (Table 3).

### 2.9. Accuracy

**Figure 7:**
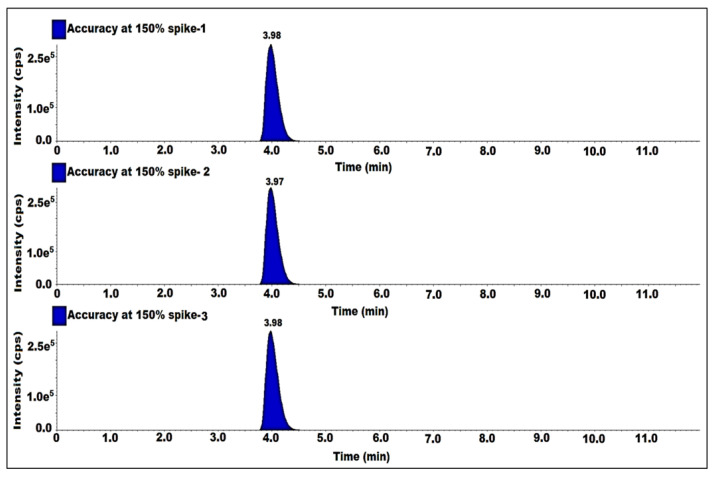
MS/MS MRM chromatogram of accuracy at 150% solutions.

The accuracy was established by spiking the impurity into a sitagliptin sample in the range of the LOQ up to a concentration level of 150%. The solutions were prepared by spiking 7-nitroso impurity into the sample at LOQ, 50, 100 and 150%. Each level was prepared in triplicate and each level was given a single injection. Determined the %recovery of impurity content from spiked sample solutions. The %recovery was observed between 80% and 120% for all the recovery levels (Table 3). Hence, the method was accurate (Figure 7).

### 2.10. Robustness

The robustness parameter was used to confirm the ability of the method when slight changes were applied to the final method. The column flow rate was changed to a plus (+) flow of 0.7 mL/min, a minus (−) flow of 0.5 mL/min and the column oven temperature changed to a plus (+) column oven temperature at 42 °C and minus (−) column oven temperature at 38 °C. The results were compared with a standard and spike solution at specification levels of MP for RT and a concentration of 7-nitroso impurity. The % difference between the impurity content obtained in the method precision and robustness study was less than 10% and the variation in the retention time of the analyte was ≤0.5 min (Table 3).

### 2.11. Solution Stability

Stability studies were performed using a secondary intermediate stock solution of 7-nitroso impurity and spiked samples with 7-nitroso impurity at 100% concentration levels up to 48 h at ambient laboratory temperatures (25 ± 5 °C) and refrigerated conditions (2–8 °C). The percentage recovery for primary standard solutions of 7-nitroso impurity and spiked samples subjected to stability studies were calculated by comparing them against the freshly prepared primary standard solutions of 7-nitroso impurity (Table 3).

Liquid chromatography with tandem mass spectrometry is a powerful analytical technique for the highly specific and quantitative measurement of very low levels of analytes and impurities in the pharmaceutical industry. An optimized LC–MS/MS method was developed to determine the 7-nitroso impurity content in sitagliptin drug substances. Since the molecular mass is specific for each compound and impurity, no interference was observed at the retention time of the impurity due to other drug substances or blanks. An advantage of this method is its ability to detect 7-nitroso impurity at ppm to ppb levels whereas the reported methods [5,6,7,8,9,10,11,12,13,14,15,16] such as the SFC-MS/MS method, RP-HPLC method, HPTLC, HPLC method, HPLC-CD, LC-UV, LC-MS and FT-IR methods, and the UV spectrophotometric and UPLC-MS/MS methods are unable to determine the content of the 7-nitroso impurity. The developed method is simple and direct and no other derivatization process is required. This method has the following advantages over the other reported methods. While detection using LC–MS/MS would be a more sensitive and reproducible approach, the proposed method shows high accuracy and precision, as indicated by the results of the validation study. The sensitivity was evaluated by the limit of quantification. The LOQ was determined to be 0.005 ppm. This method is as good or superior to those reported in other papers.

Additional validation chromatograms and standard qualification data are available in the Supplementary Materials (Figures S1–S21).

## 3. Materials and Methods

### 3.1. Materials and Reagents

In this study, 7-nitroso impurity and sitagliptin phosphate monohydrate were procured from Jisai Pharma Pvt Ltd. Plot No.12, Phase-4, IDA Cherlapally, Hyderabad—500051, (India). Formic acid and methanol were procured from Fischer Chemicals and Carlo Erba Chemicals, (India). Water of HPLC grade was procured from Rankem^®^ and used for the preparation of all buffers and standard solutions.

### 3.2. Equipment

A UHPLC system connected to a triple quadrupole QTRAP MS/MS equipped with an electrospray ionization (ESI) probe (ABsciex QTRAP 4500) was used for the method development and validation. Data analyzing software was used to collect and analyze the data. A Mettler Toledo analytical balance was used for standards and weighing samples.

### 3.3. Chromatographic Conditions

The chromatographic conditions were finalized by considering both analytes, based on the method development data. The quantification of the compound was achieved with a C18 column (100 × 4.6 mm, 3.5 µm particle size) at an oven temperature of 40 °C. The mobile phase was composed of 0.12% formic acid in water, with methanol as a mobile phase A and B, and the flow rate was set at 0.6 mL/min and the “gradient elution program” was deployed, which gave the best response within an acceptable analysis time and column back pressure. The injection volume was 50 µL.

### 3.4. Mass Spectrometer Conditions

The MS/MS detector is highly sensitive and reproducible. The MS detector was operated with electrospray ionization (ESI), which utilized a positive ion source and multiple reaction monitoring (MRM) at m/z Q1 as 221.9 and Q3 as 191.9. De-clustering potential (DP 40), entrance potential (EP 10), collision energy (CE 15) and an MS temperature of 400 °C were used as the MS/MS detector conditions.

### 3.5. Preparation of Impurity Standard and Test Sample Solutions

Standard and sample concentrations were finalized by using the required dilutions, based on the response of impurities during the study by using water as diluent. We prepared a 0.03 ppm concentration of 7-nitroso impurity standard in water using the required dilutions. A sitagliptin sample of 50 mg/mL was prepared in water and sonicated for five minutes. Then, it was vortexed again for five minutes and mixed well. The sample solution was filtered by using a 0.45 µm nylon filter and water was injected as a blank.

## 4. Conclusions

The sensitive, selective, and rapid USPLC LC-MS/MS method developed for the identification and quantification of 7-nitroso impurity in sitagliptin phosphate monohydrate is able to detect impurity at trace level concentrations. Thus, this new method, which uses advanced technology, is capable of identifying and detecting 7-nitroso impurity at ppm and ppb levels. Considering the industrial requirements and guidelines, the method was validated in line with the ICH and USP. The method is specific, linear, precise, accurate and robust. The results obtained by using this method demonstrated that reliable data can be obtained in further experiments, for example, in relation to sitagliptin drug substances and drug products.

## Figures and Tables

**Table 1:** Liquid chromatographic and mass spectrometric method conditions.

Parameter	Condition
**Liquid chromatography conditions**	
Mobile phase A	0.12% formic acid in water
Mobile phase B	0.12% formic acid in Methanol
Auto-sampler temperature	8 °C
Temperature of the column	40 °C
Flow rate	0.6 mL/min
Injection volume	50 μL
Gradient program (time(min)/mobile phase A)	0/75, 6/75, 6.1/5, 8/5, 8.1/75, 12/75
Diluent	Water
Run time	12 min
**Mass spectrometry conditions**	
Source and ionization mode	ESI-Positive
Detection mode	MRM
MRM (m/z) for quantification	221.9 > 191.9
Collision energy (CE)	15
De-clustering potential (DP)	40
Entrance potential (EP)	10
MS temperature	400 °C

**Table 2:** Summary of results of the method validation.

Validation Parameter	Typical Acceptance Criteria	Results
System suitability and Specificity	RSD (%) for 7-nitroso impurity peak area response (*n* = 6) should be ≤ 15.0.	1.1%
	RT of 7-nitroso impurity peak in all the solutions.	4.0 min
	Interference from blank	No interference
LOD	Concentration of LOD in ppm s/n value should be ≥3	0.002 ppm 11
LOQ	Concentration of LOQ in ppm s/n value should be ≥10	0.005 ppm 33
LOQ precision	RSD (%) for six replicate injections of LOQ solution should be ≤ 15.0%	3.1%
Linearity	Range (ppm) Square of correlation coefficient (r^2^) ≥ 0.99	0.005 to 0.06 ppm 0.999

**Table 3:** Summary of method validation results.

Validation Parameter	Typical Acceptance Criteria	Results
Method precision	RSD (%) for six preparations (*n* = 6) of spiked sample at specification level should be ≤10.0	2.8%
Intermediate precision	RSD (%) for six preparations (*n* = 6) of spiked sample at specification level should be ≤10.0	3.2%
	RSD (%) for preparations (*n* = 12) of MP and IP spiked sample at specification level should be ≤20.0	Less than 20.0%
Accuracy	LOQ average recovery (*n* = 3) should be between 70 to 130%.	92.1%
	50% average recovery (*n* = 3) should be between 80 to 120%.	89.6%
	100% average recovery (*n* = 3) should be between 80 to 120%.	95.2%
	150% average recovery (*n* = 3) should be between 80 to 120%.	97.3%
Robustness	Plus (+) flow 0.7 mL/min: spiked sample concentration % difference and retention time	2.3% 3.7 min
	Minus (−) flow 0.5 mL/min: spiked sample concentration % difference and retention time	2.5% 4.1 min
	Plus (+) oven 42 °C: spiked sample concentration % difference and retention time	2.1% 3.8 min
	Minus (−) oven 38 °C: spiked sample concentration % difference and retention time	2.7% 4.0 min
Solution Stability	Standard and 100% spiked solution stored at ambient laboratory conditions (25 ± 5 °C) and refrigerated conditions (2–8 °C) were studied for 48 h	Solutions are Stable for 48 h

## Data Availability

Data are contained within the article and in the Supplementary Materials.

## Supplementary Materials

The following supporting information can be downloaded at: https://www.mdpi.com/article/10.3390/molecules27238581/s1, Figure S1: MS/MS chromatogram of Blank solution; Figure S2: MS/MS chromatogram of 7-nitroso impurity spiked sample; Figure S3: MS/MS chromatogram of System suitability standard solution; Figure S4: MS/MS chromatogram of Sitagliptin sample solution; Figure S5: MS/MS chromatogram of LOD solution; Figure S6: MS/MS chromatogram of LOQ solution; Figure S7: MS/MS chromatograms of LOQ Precision; Figure S8: MS/MS chromatograms of Linearity solution; Figure S9: MS/MS chromatograms of Method precision; Figure S10: MS/MS chromatograms of Intermediate precision; Figure S11: MS/MS chromatograms of LOQ Accuracy; Figure S12: MS/MS chromatograms of LOQ Accuracy; Figure S13: MS/MS chromatograms of Standard solution stability; Figure S14: MS/MS chromatograms of Spiked sample solution stability; Figure S15: 7-nitroso impurity Certificate of analysis; Figure S16: 7-nitroso impurity purity by HPLC chromatogram; Figure S17: 7-nitroso impurity Mass spectrum; Figure S18: 7-nitroso impurity 1H NMR spectrum—1; Figure S19: 7-nitroso impurity 1H NMR spectrum—2; Figure S20: 7-nitroso impurity TGA graph; Figure S21: Probable Fragmentation pattern of 7-nitroso impurity in the mass ionization chamber due to parsing pyrolysis.

## Sample Availability

Samples of the compounds are available on request from the authors.

## References

[1] Szekely G., Amores de Sousa M.C., Gil M., Castelo Ferreira F., Heggie W. (2015). Genotoxic impurities in pharmaceutical manufacturing: Sources, regulations, and mitigation. Chem. Rev..

[2] Reddy A.V.B., Jaafar J., Umar K., Majid Z.A., Aris A.B., Talib J., Madhavi G. (2015). Identification, control strategies, and analytical approaches for the determination of potential genotoxic impurities in pharmaceuticals: A comprehensive review. J. Sep. Sci..

[3] Ahuja S. (2000). Isolation and Characterization of Pharmaceutical Impurities Evaluation.

[4] Wang T., Yang H., Yang J., Guo N., Wu G., Xu X., An M. (2022). Quantitative Determination of Four Potential Genotoxic Impurities in the Active Pharmaceutical Ingredients in TSD-1 Using UPLC-MS/MS. Molecules.

[5] Pokar D., Rajput N., Sengupta P. (2020). Industrial approaches and consideration of clinical relevance in setting impurity level specification for drug substances and drug products. Int. J. Pharm..

[6] Zeller A., Brigo A., Brink A., Guerard M., Lang D., Muster W., Runge F., Sutter A., Vock E., Wichard J. (2019). Genotoxicity assessment of drug metabolites in the context of MIST and beyond. Chem. Res. Toxicol..

[7] Zhang K., Pellett J.D., Narang A.S., Wang Y.J., Zhang Y.T. (2018). Reactive impurities in large and small molecule pharmaceutical excipients–A review. TrAC Trends Anal. Chem..

[8] Guideline I.H.T. Impurities in new drug substances Q3A (R2). Proceedings of the International Conference on Harmonization of Technical Requirements for Registration of Pharmaceuticals for Human Use.

[9] Guideline I. Assessment and control of dna reactive (mutagenic) impurities in pharmaceuticals to limit potential carcinogenic risk M7. Proceedings of the International Conference on Harmonization of Technical Requirements for Registration of Pharmaceuticals for Human Use (ICH).

[10] Gao H., Yu J., Ge C., Jiang Q. (2018). Practical asymmetric synthesis of sitagliptin phosphate monohydrate. Molecules.

[11] US Food and Drug Administration label Januvia of Merck. https://www.accessdata.fda.gov/drugsatfda_docs/label/2012/021995s019lbl.pdf.

[12] Scott L.J. (2017). Sitagliptin: A review in type 2 diabetes. Drugs.

[13] (2020). Rev.11 of Questions and Answers for Marketing Authorisation Holders/Applicants on the CHMP Opinion for the Article 5(3) of Regulation (EC) No 726/2004 Referral on Nitrosamine Impurities in Human Medicinal Products.

[14] Schmidtsdorff S., Neumann J., Schmidt A.H., Parr M.K. (2022). Risk assessment for nitrosated pharmaceuticals: A future perspective in drug development. Arch. Der Pharm..

[15] Vuyyuru N.R., Krishna G.V., Ramadevi B., Kumar Y.R. (2017). Evaluation of Process Impurities and Degradants of Sitagliptin Phosphate by Validated Stability Indicating RP-LC Method. Asian J. Chem..

[16] Deepthi V., Poornima Y., Rao G.D., Reddy T.S. (2013). Stability-indicating RPHPLC method for analysis of sitagliptin in the bulk drug and it’s pharmaceutical dosage form. Int. J. Pharm. Pharm. Sci..

[17] Jain P., Chaudhari A., Surana S. (2014). Development and validation of stability-indicating high-performance thin-layer chromatography method for estimation of sitagliptin phosphate monohydrate in bulk and in pharmaceutical formulation. Acta Chromatogr..

[18] Saleh O.A., El-Azzouny A.A.E.-S., Aboul-Enein H.Y., Badawey A.M. (2014). A validated stability indicating HPLC method for determination of sitagliptin. Eur. J. Chem..

[19] Ramalingam P., Bhaskar V.U., Reddy Y.P., Kumar K.V. (2014). Stability-indicating RP-HPLC method for the simultaneous determination of sitagliptin and simvastatin in tablets. Indian J. Pharm. Sci..

[20] Dalawai M.V., Sanasi P.D., Sharma H.K. (2016). Development and Validation of Simple Effective Hplc Method for the Quantitative Determination of Related Substance Present in Sitagliptin Phosphate Drug Substance. Int. J. Pharm. Sci. Res..

[21] Dalawai M.V., Sanasi P., Sharma H. (2015). Development and validation of stability indicating assay method by HPLC for the analysis of sitagliptin phospahte in bulk drug substances. J. Chem. Pharm. Res..

[22] Kirkpatrick D., Fain M., Yang J., Trehy M. (2018). Enantiomeric impurity analysis using circular dichroism spectroscopy with United States Pharmacopeia liquid chromatographic methods. J. Pharm. Biomed. Anal..

[23] Gumieniczek A., Berecka A., Mroczek T., Wojtanowski K., Dąbrowska K., Stępień K. (2019). Determination of chemical stability of sitagliptin by LC-UV, LC-MS and FT-IR methods. J. Pharm. Biomed. Anal..

[24] Pathade P. (2011). Stability indicating UV Spectrophotometric method has been developed for quantitative determination of Sitagliptin Phosphate in bulk and pharmaceutical formulations. J. Pharm. Res..

[25] Mowaka S., Mohamed D. (2015). Novel contribution to the simultaneous analysis of certain hypoglycemic drugs in the presence of their impurities and degradation products utilizing UPLC-MS/MS. RSC Adv..

[26] Guideline I.H.T. (2005). Validation of Analytical Procedures: Text and Methodology. Q2.

