# Supplementary material for: Development and Validation for Quantification of 7-Nitroso Impurity in Sitagliptin by Ultraperformance Liquid Chromatography with Triple Quadrupole Mass Spectrometry
Source: Molecules. 2022 Dec 5;27(23):8581. doi: 10.3390/molecules27238581 (PMC9736263; doi:10.3390/molecules27238581)
Supplement: Supplementary file 1 [file molecules-27-08581-s001.zip › molecules-2039406-supplementary.pdf]

Supplementary File

Development and Validation for Quantification of 7-Nitroso Impurity in Sitagliptin by Ultraperformance Liquid Chromatography with Triple Quadrupole Mass Spectrometry

Hari Naga Prasada Reddy Chittireddy [1], J. V. Shanmukha Kumar [1,*], Anuradha Bhimireddy [2], Mohammed Rafi Shaik [3,*], Merajuddin Khan [3], Syed Farooq Adil [3], Mujeeb Khan [3] and Fatimah N. Aldhuwayhi [3]

[1] Department of Engineering Chemistry, College of Engineering, Koneru Lakshmaiah Education Foundation, Vaddeswaram, Guntur 522502, Andhra Pradesh, India
[2] Aurobindo Pharma Limited, Sanga Reddy, Indrakaran 502329, Telangana, India
[3] Department of Chemistry, College of Science, King Saud University, P.O. Box 2455, Riyadh 11451, Saudi Arabia
* Correspondence: shanmukh_fed@kluniversity.in (J.V.S.K.); mrshaik@ksu.edu.sa (M.R.S.); Tel.: +966-11-4670439 (M.R.S.)

Validation parameters:

1. **System suitability and Specificity chromatograms:**

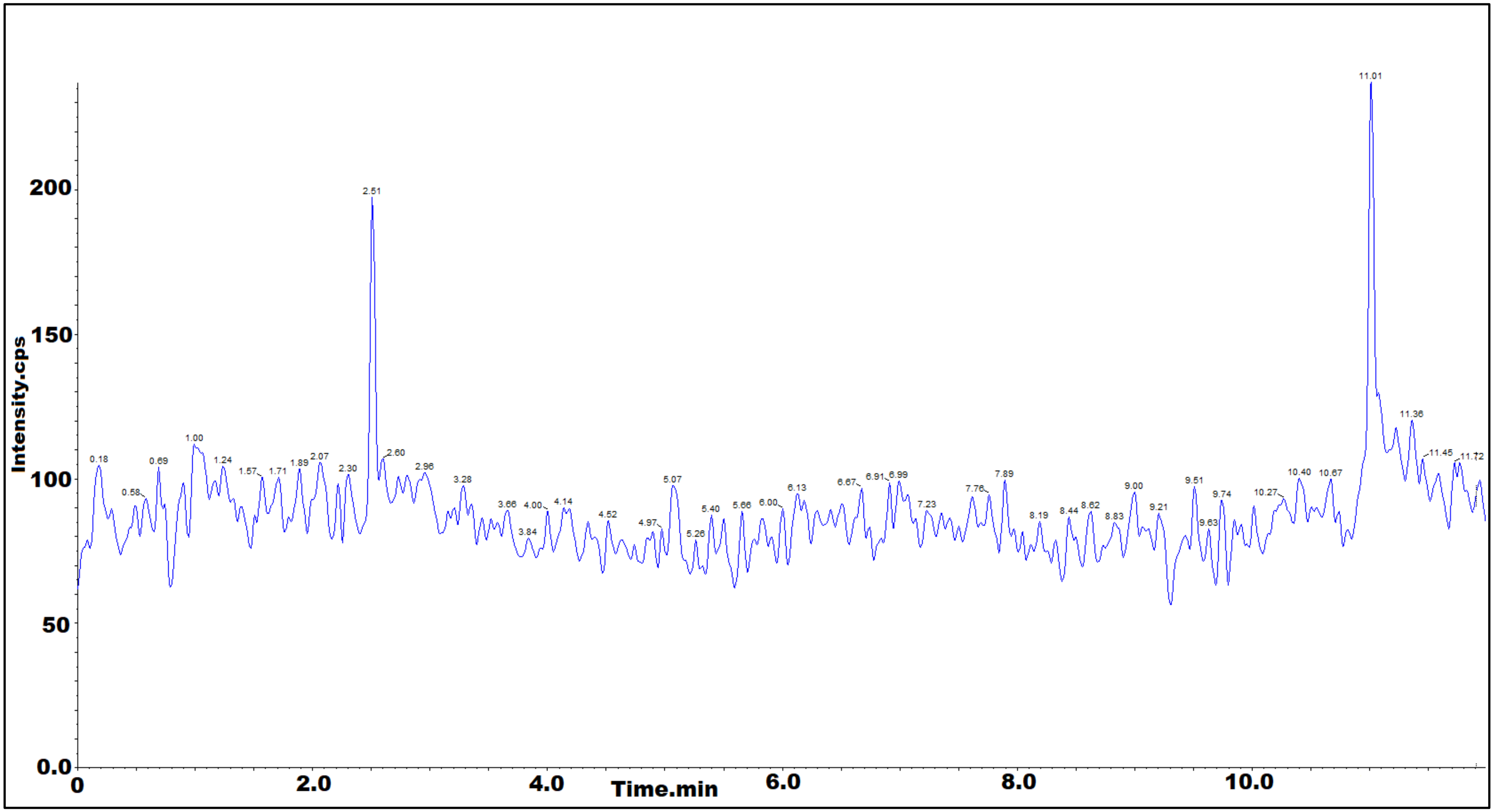

Figure S1: MS/MS chromatogram of Blank solution.

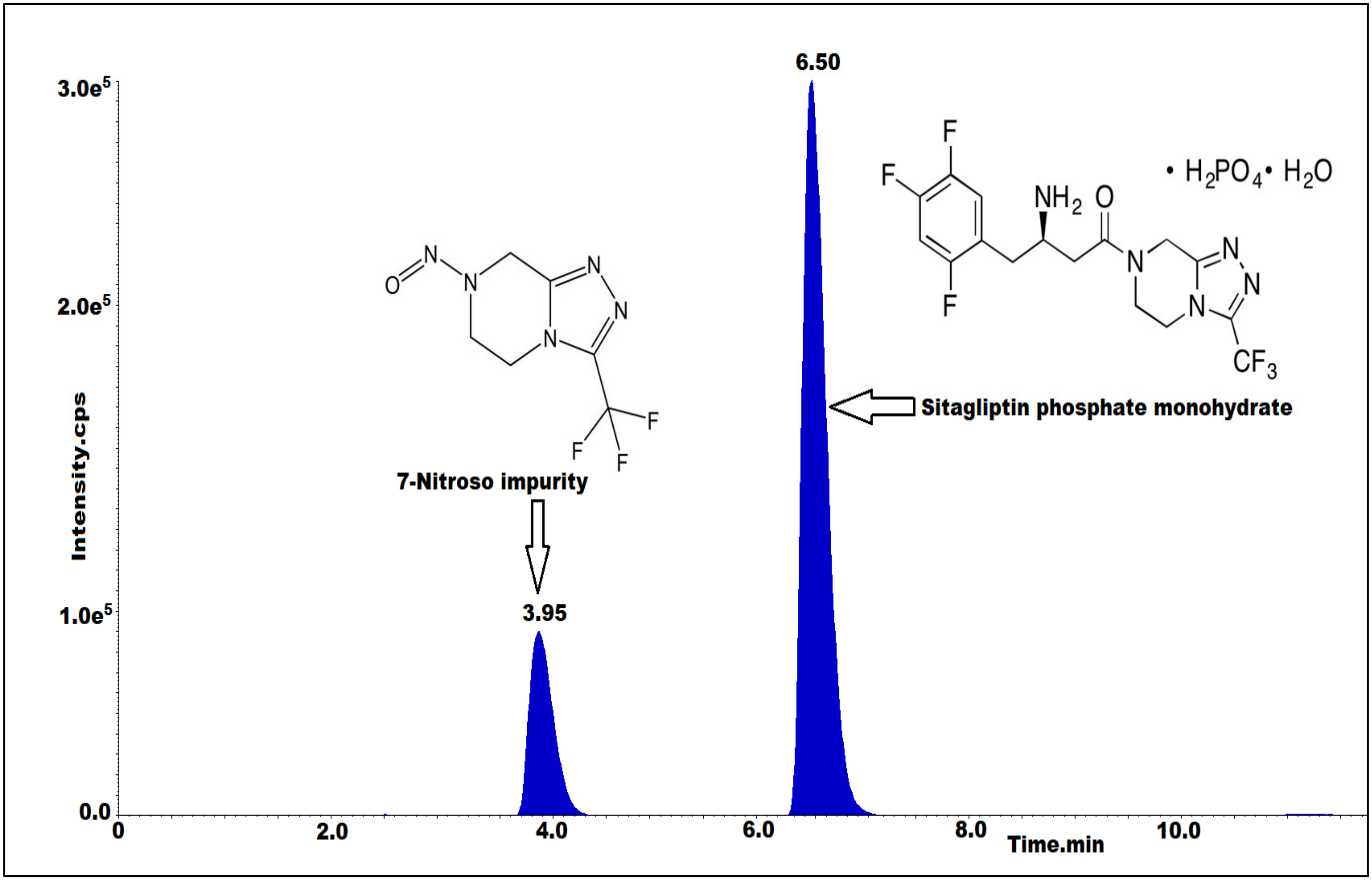

Figure S2: MS/MS chromatogram of 7-nitroso impurity spiked sample.

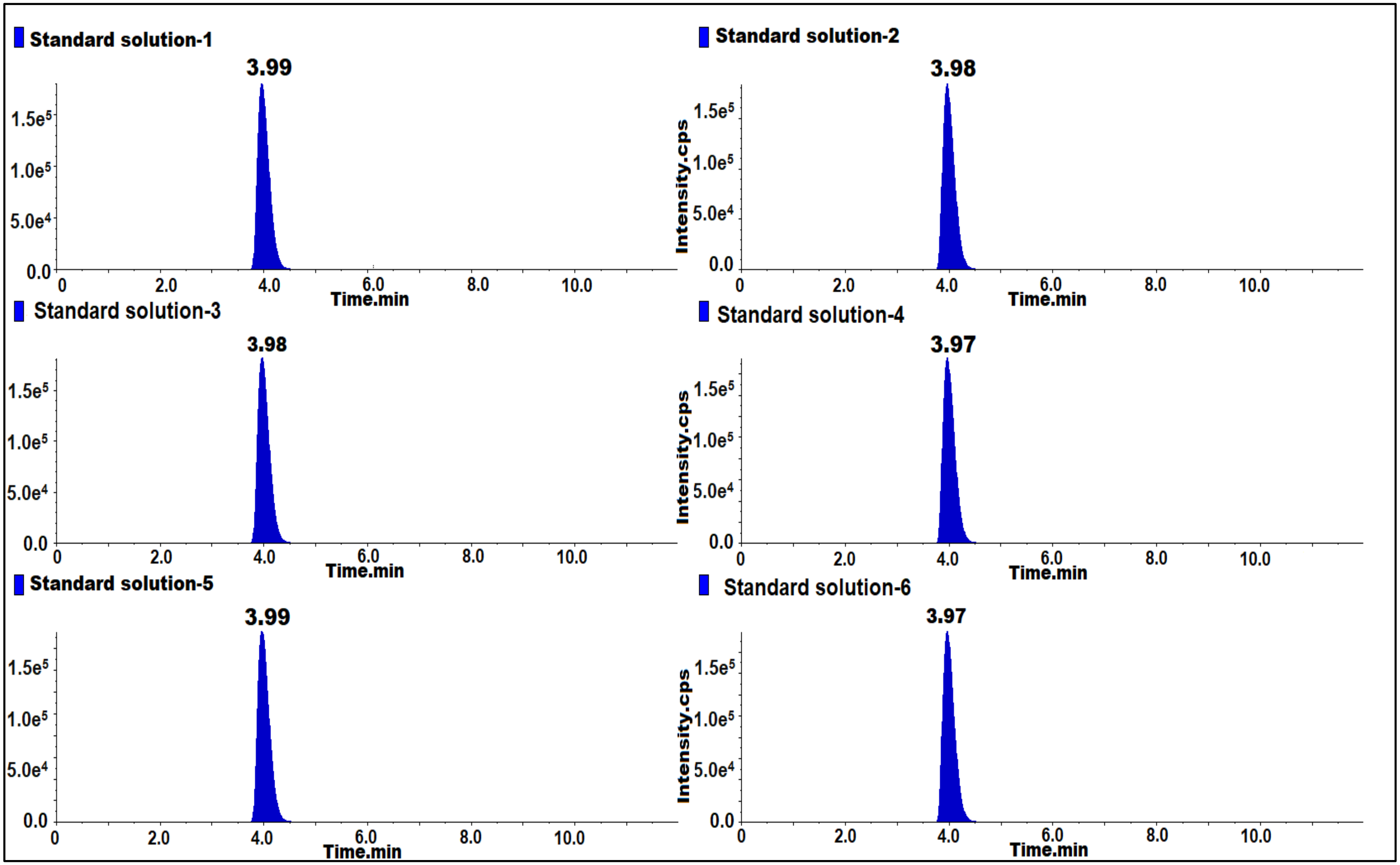

Figure S3: MS/MS chromatogram of System suitability standard solution.

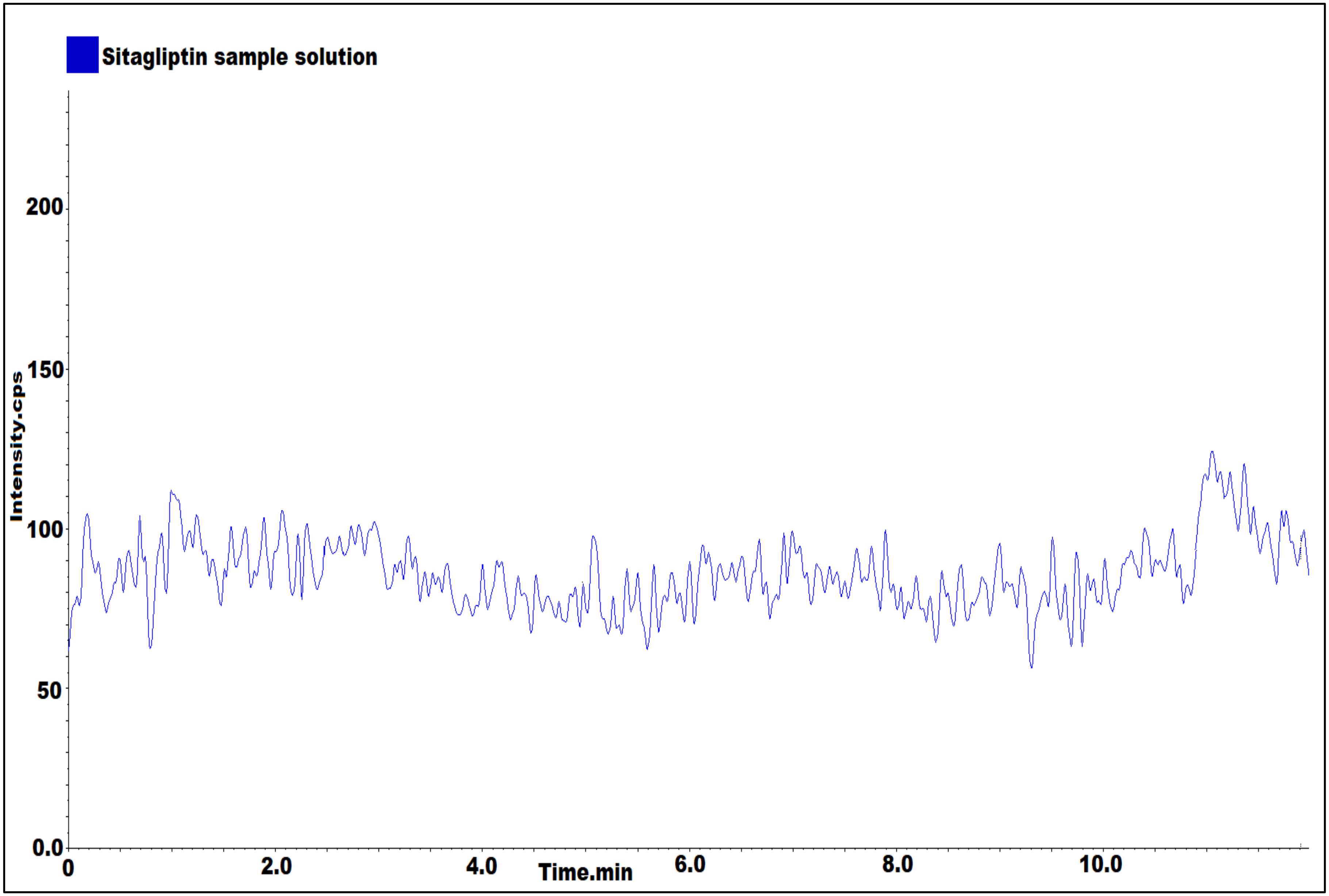

Figure S4: MS/MS chromatogram of Sitagliptin sample solution.

2. LOD, LOQ and LOQ precision

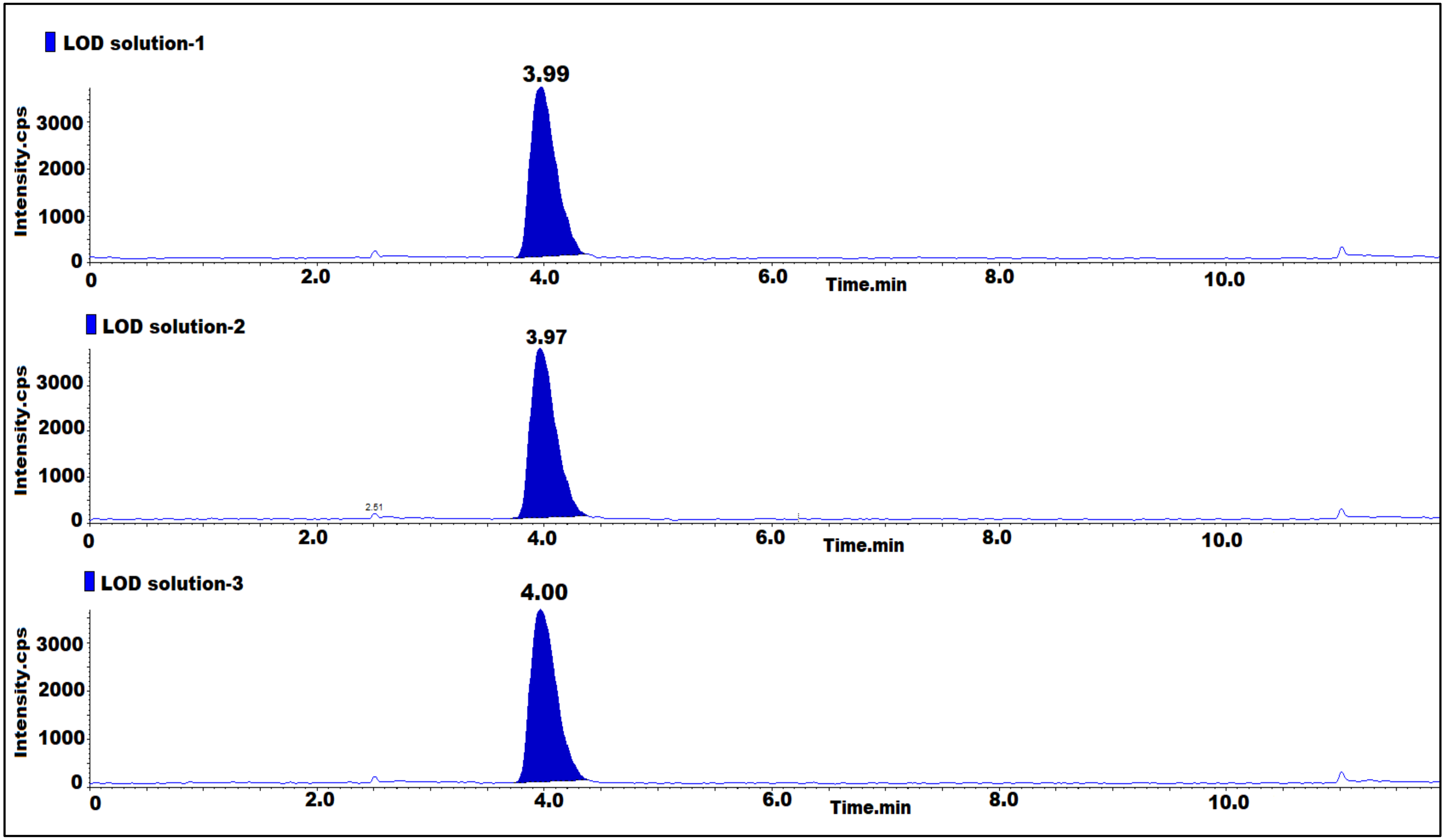

Figure S5: MS/MS chromatogram of LOD solution.

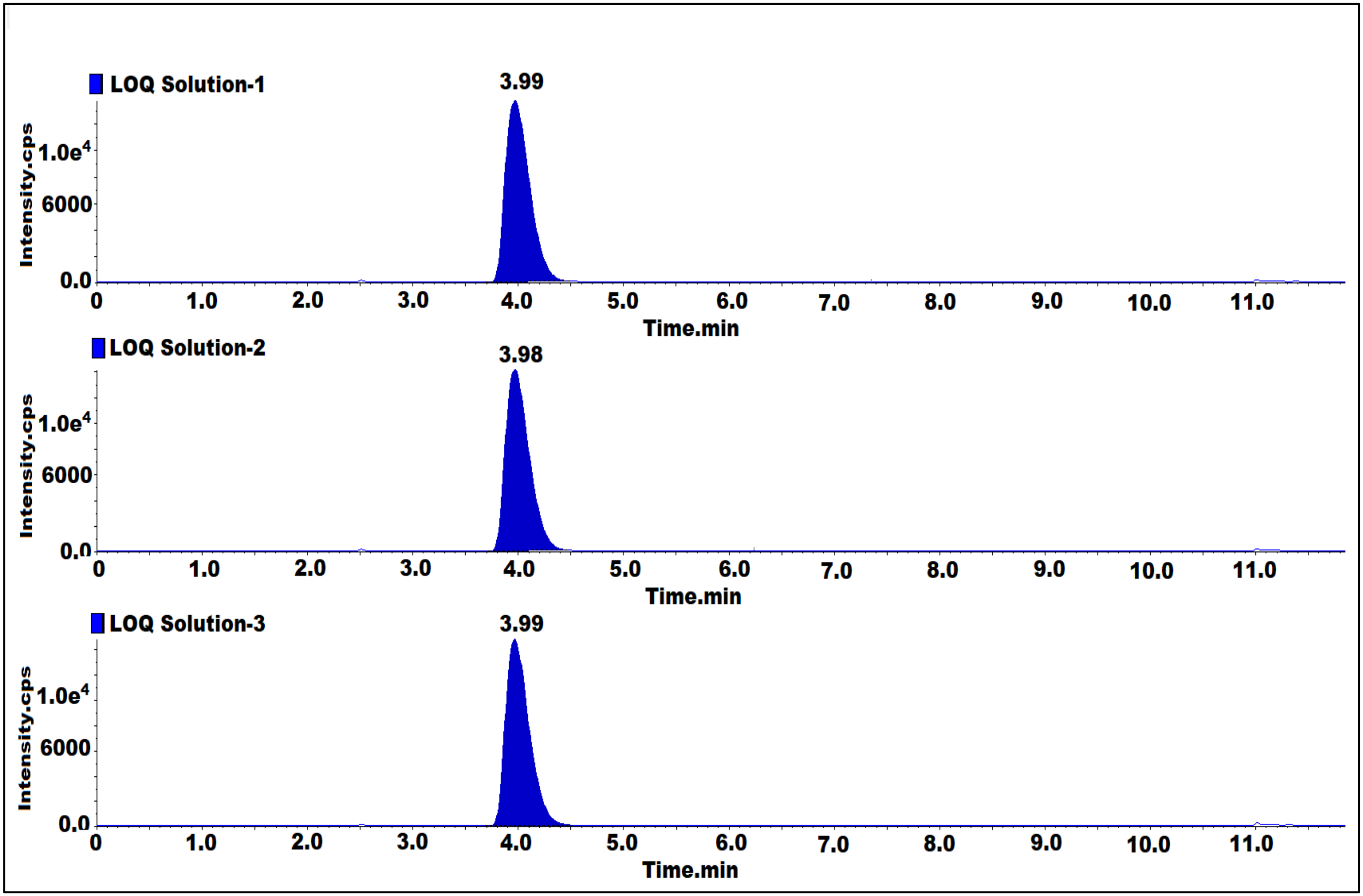

Figure S6: MS/MS chromatogram of LOQ solution.

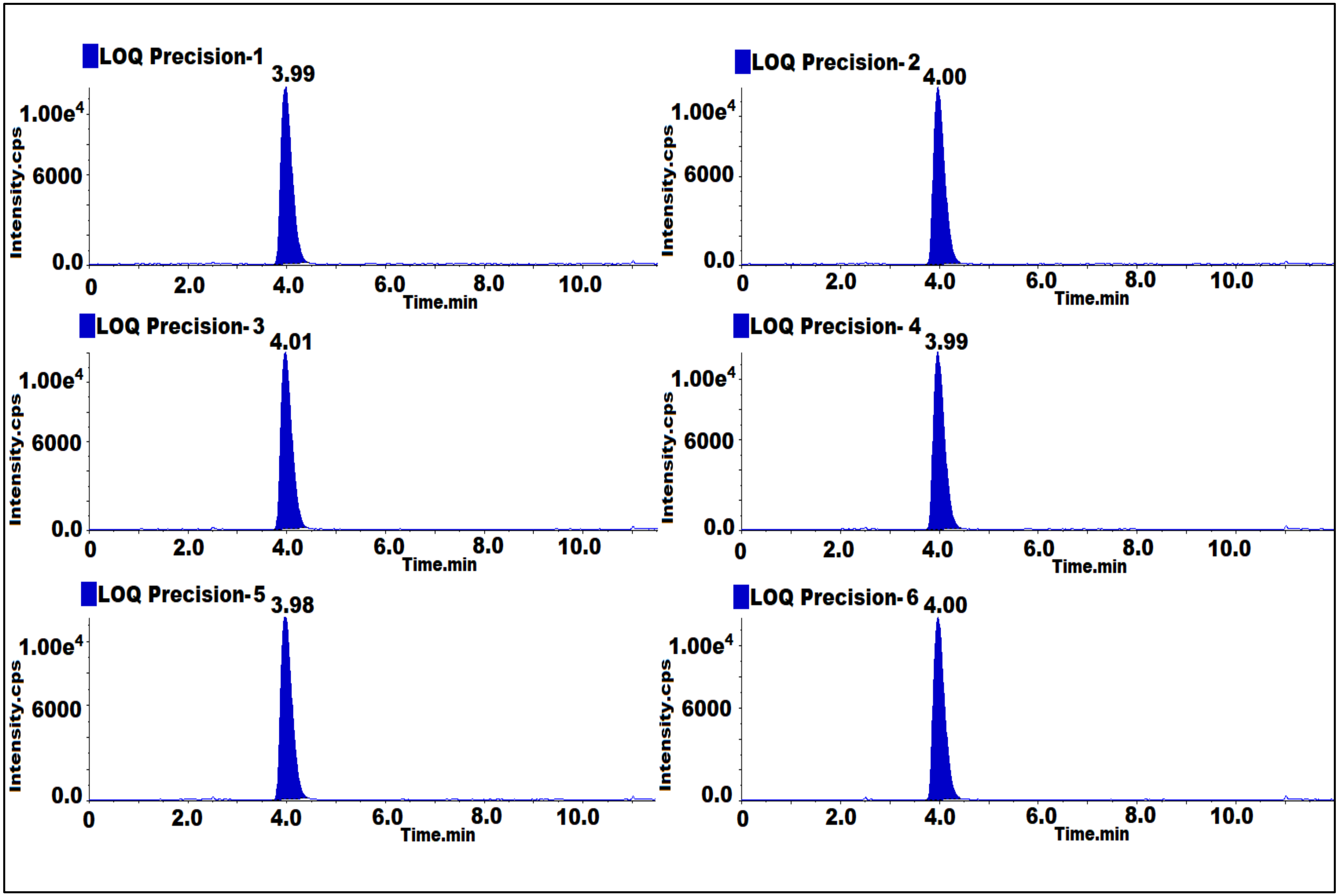

Figure S7: MS/MS chromatograms of LOQ Precision.

3. Linearity

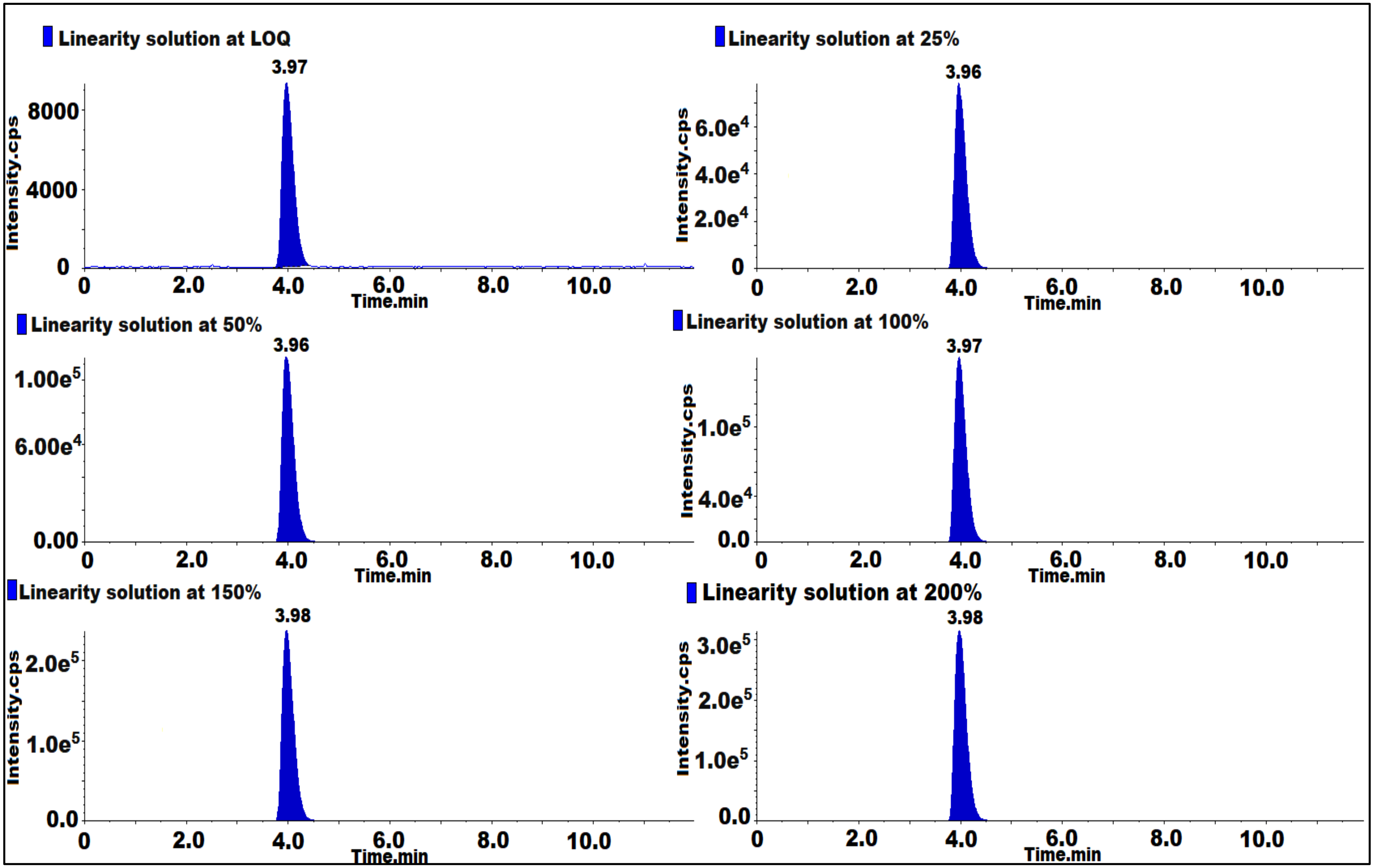

Figure S8: MS/MS chromatograms of Linearity solution.

4. Method precision

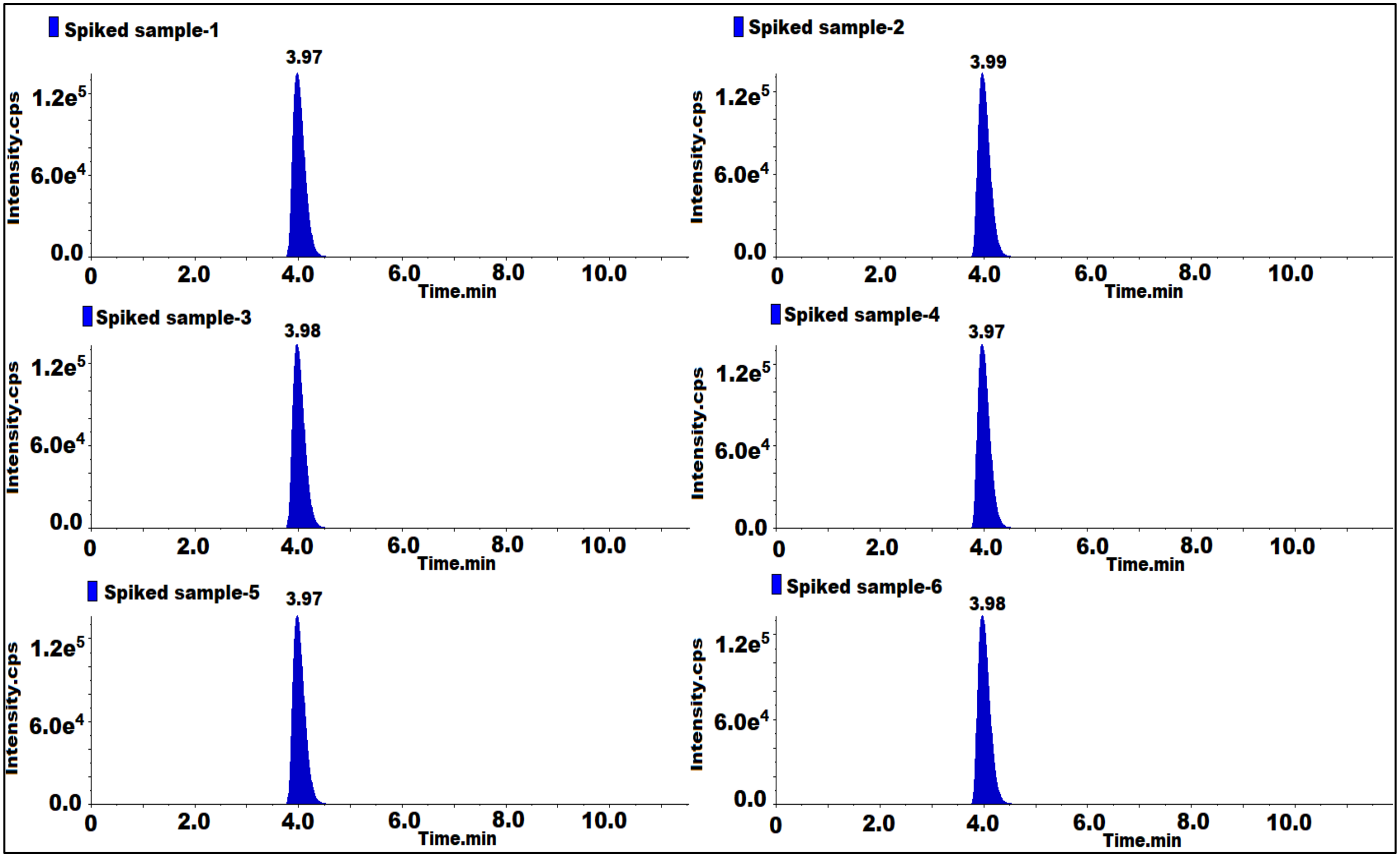

Figure S9: MS/MS chromatograms of Method precision.

5. **Intermediate precision**

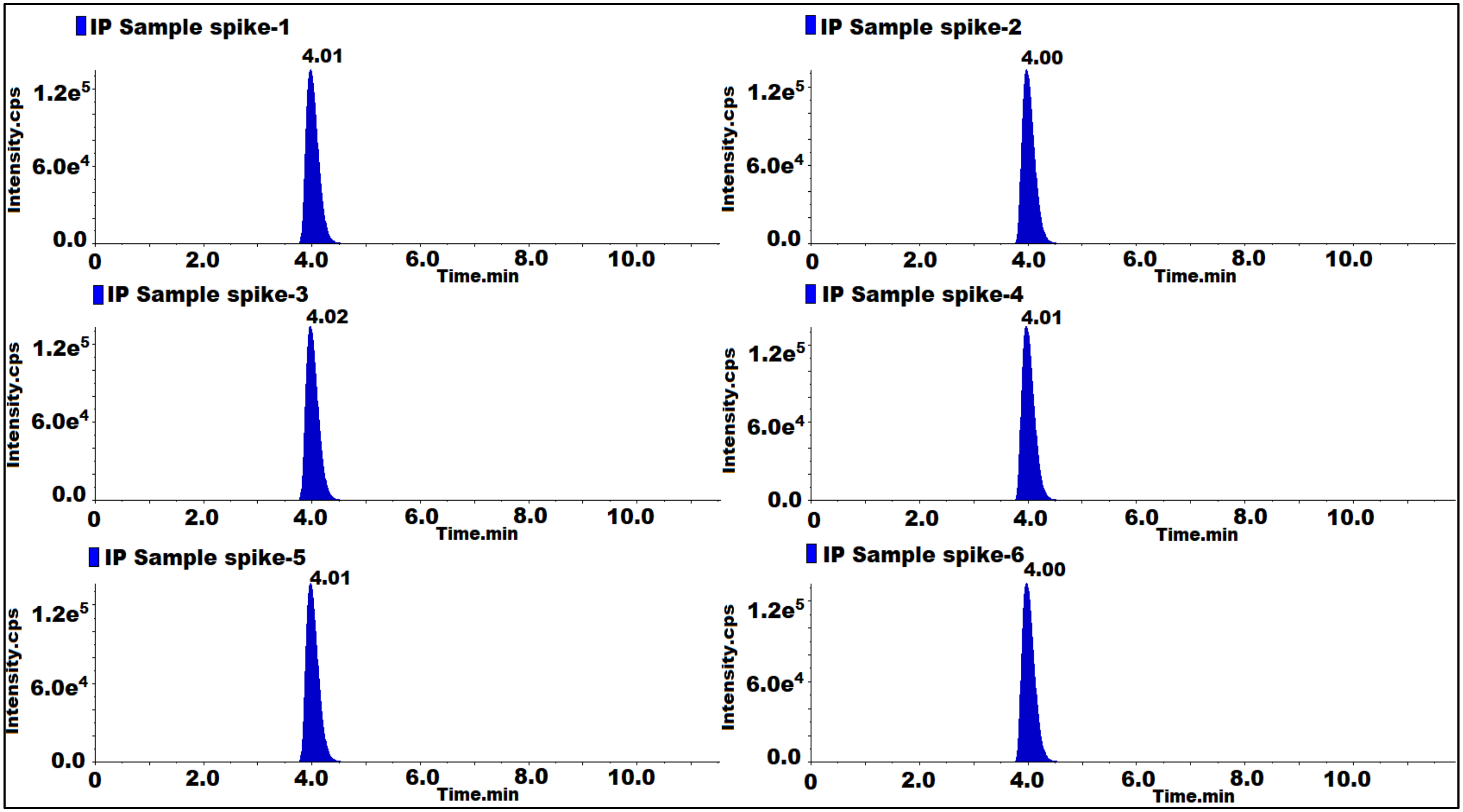

Figure S10: MS/MS chromatograms of Intermediate precision.

6. Accuracy

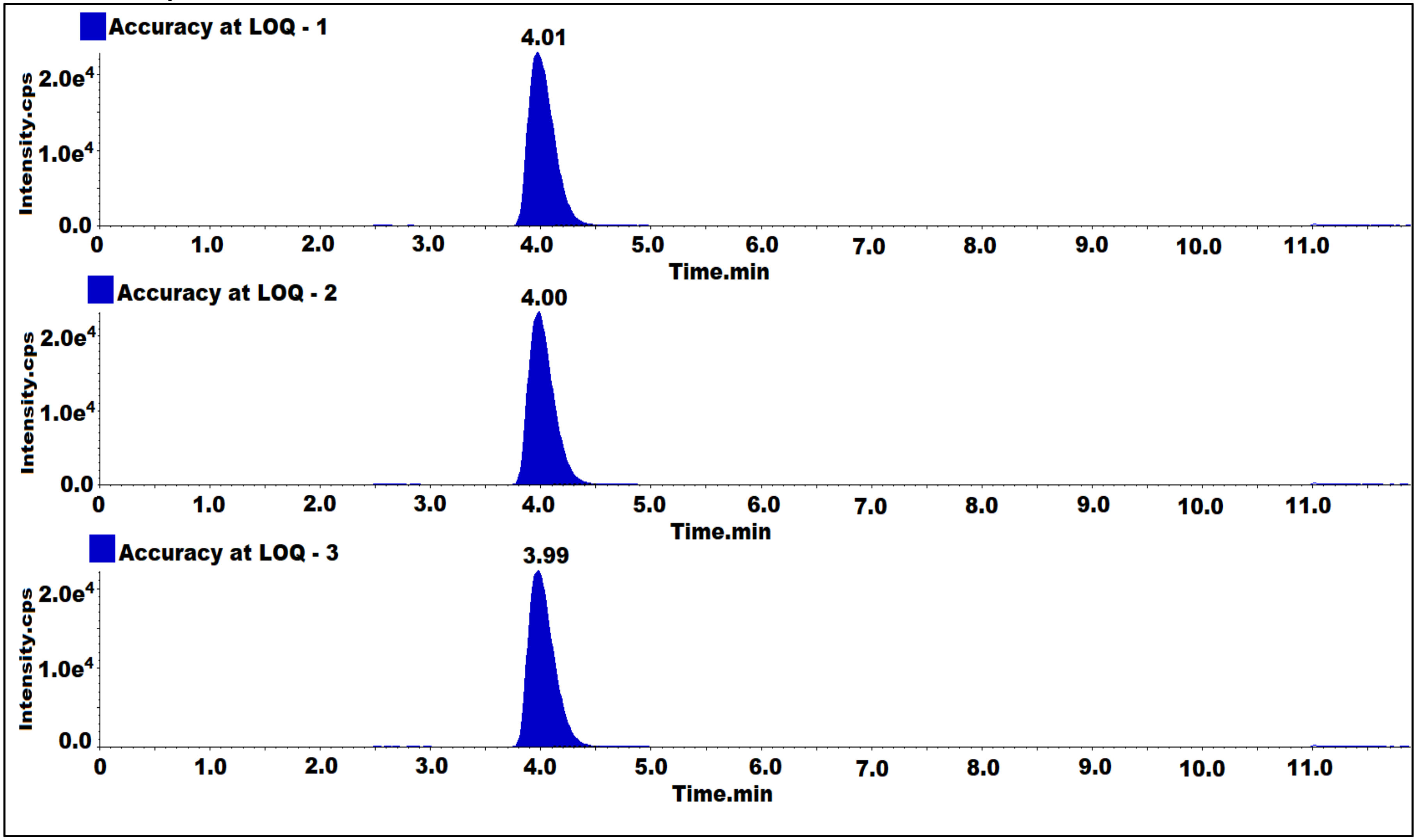

Figure S11: MS/MS chromatograms of LOQ Accuracy.

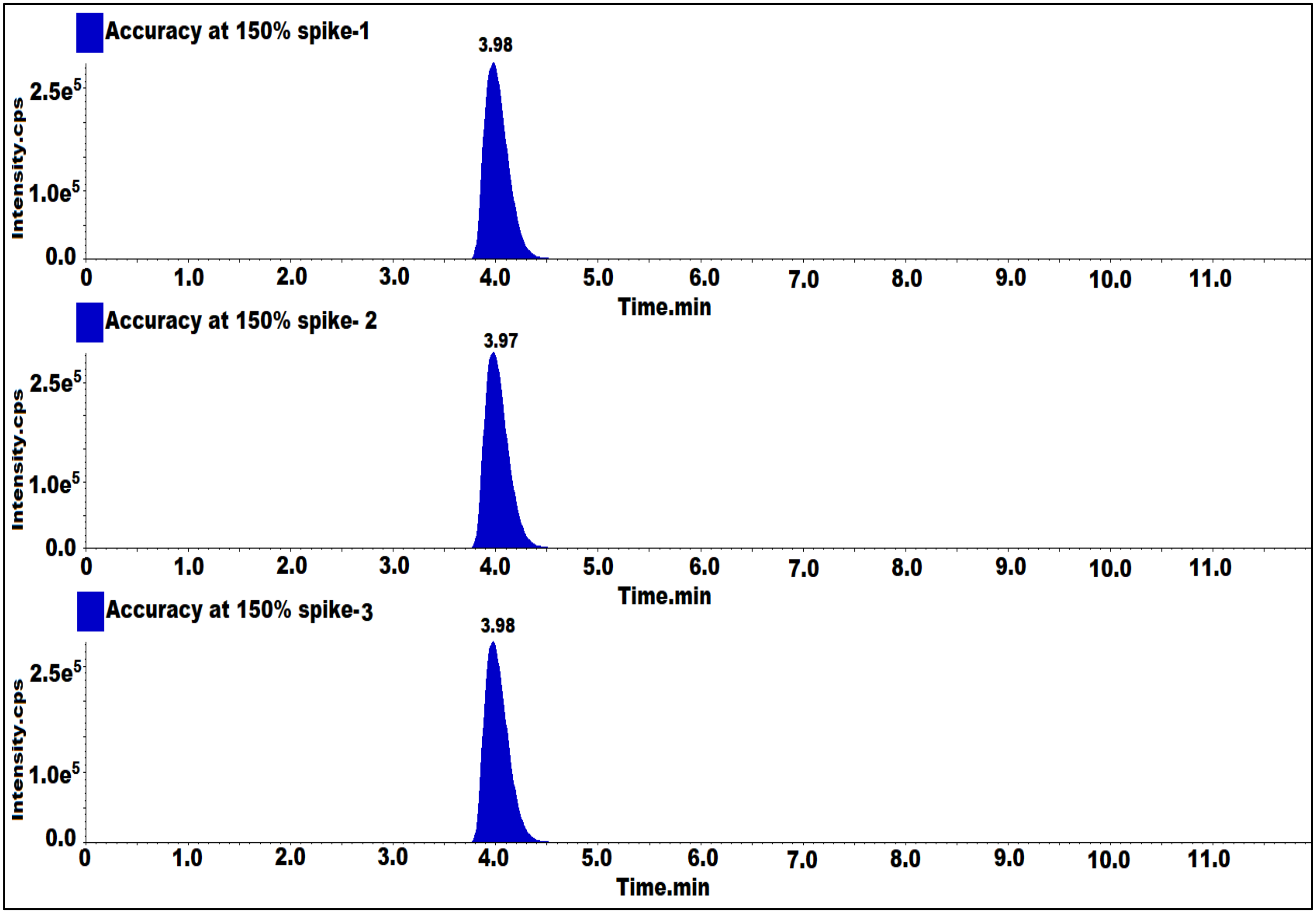

Figure S12: MS/MS chromatograms of LOQ Accuracy.

7. Solution stability

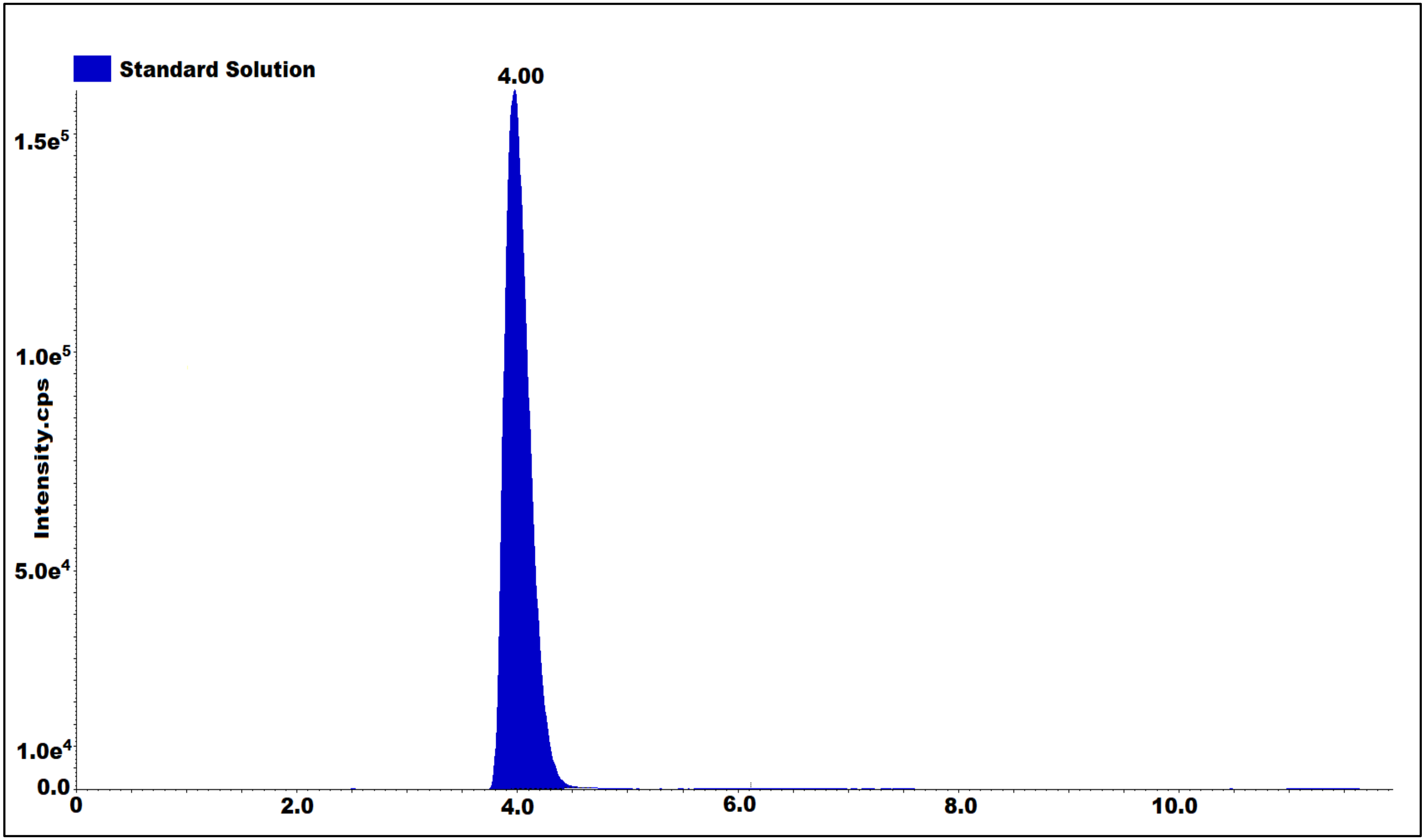

Figure S13: MS/MS chromatograms of Standard solution stability.

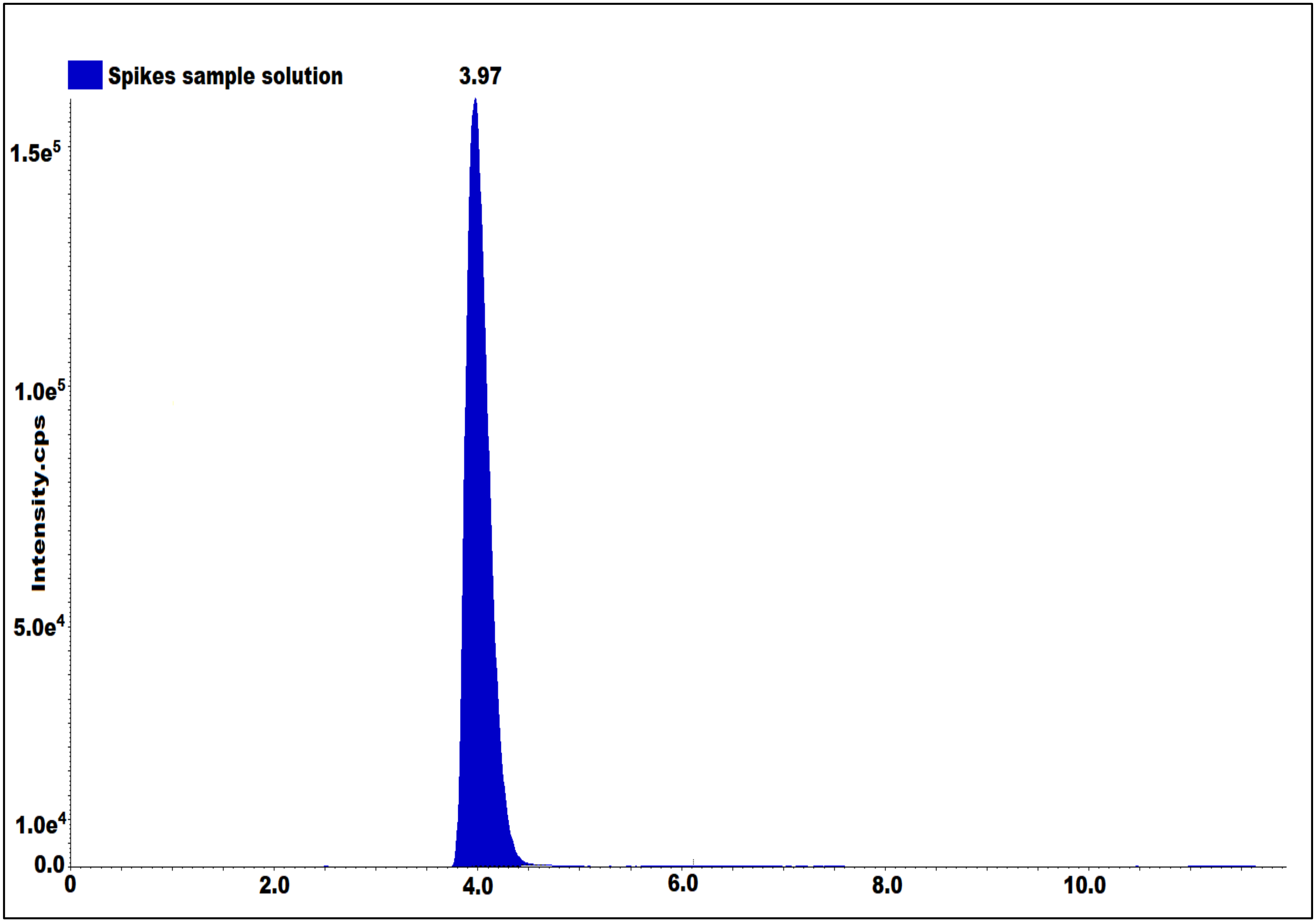

Figure S14: MS/MS chromatograms of Spiked sample solution stability.

8. Sitagliptin 7-nitroso impurity standard Qualification data

CERTIFICATE OF ANALYSIS

**Product Name**	**7-nitroso-3-(trifluoromethyl)-5,6,7,8-tetrahydro-[1,2,4]triazolo[4,3-a]pyrazine**		
**IUPAC Name**	7-Nitroso-3-(trifluoromethyl)-5,6,7,8-tetrahydro-[1,2,4]triazolo[4,3-a]pyrazine		
**Synonyms**	Not available		
**CAS #**	Not available		
**Molecular Formula**	$C_6H_6F_3N_5O$	**Molecular weight**	221.14
**Longterm Storage Conditions**	Store at refrigerator (2-8°C) for long term storage		
**Shipping Conditions**	Product is stable to be shipped at room temperature		

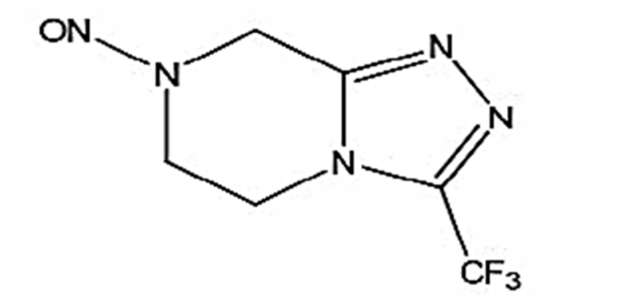

Test	Specification	Result
Appearance	Light Green Solid	Light Green Solid
Solubility	Soluble in DMSO	In DMSO
1H NMR spectrum	Conform structure	Confirms
Mass Spectrum	Conform molecular weight	Confirms
Purity by HPLC - c	Not less than 90%	99.51%
Volatiles content % (by TGA) - b	Not specified	0.50% (w/w)
Ash content % (by TGA) - a	Not specified	1.39% (w/w)
Potency based on Mass balance {[100 - (b+a)] x (c/100)}	Not specified	97.63%

Figure S15: 7-nitroso impurity Certificate of analysis.

7-nitroso impurity Purity by HPLC

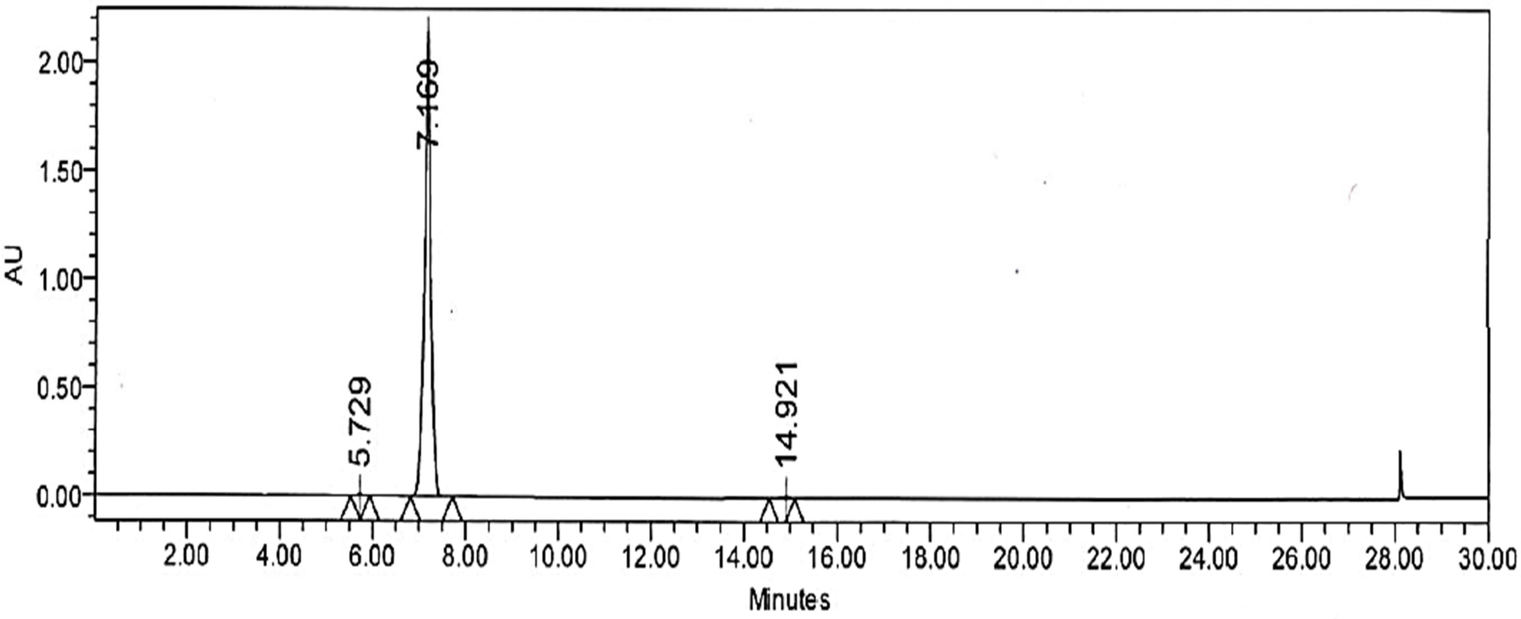

	RT	Area	% Area	Height
1	5.729	36035	0.18	7166
2	7.169	20259224	99.51	2120538
3	14.921	62885	0.31	7623

Figure S16: 7-nitroso impurity purity by HPLC chromatogram.

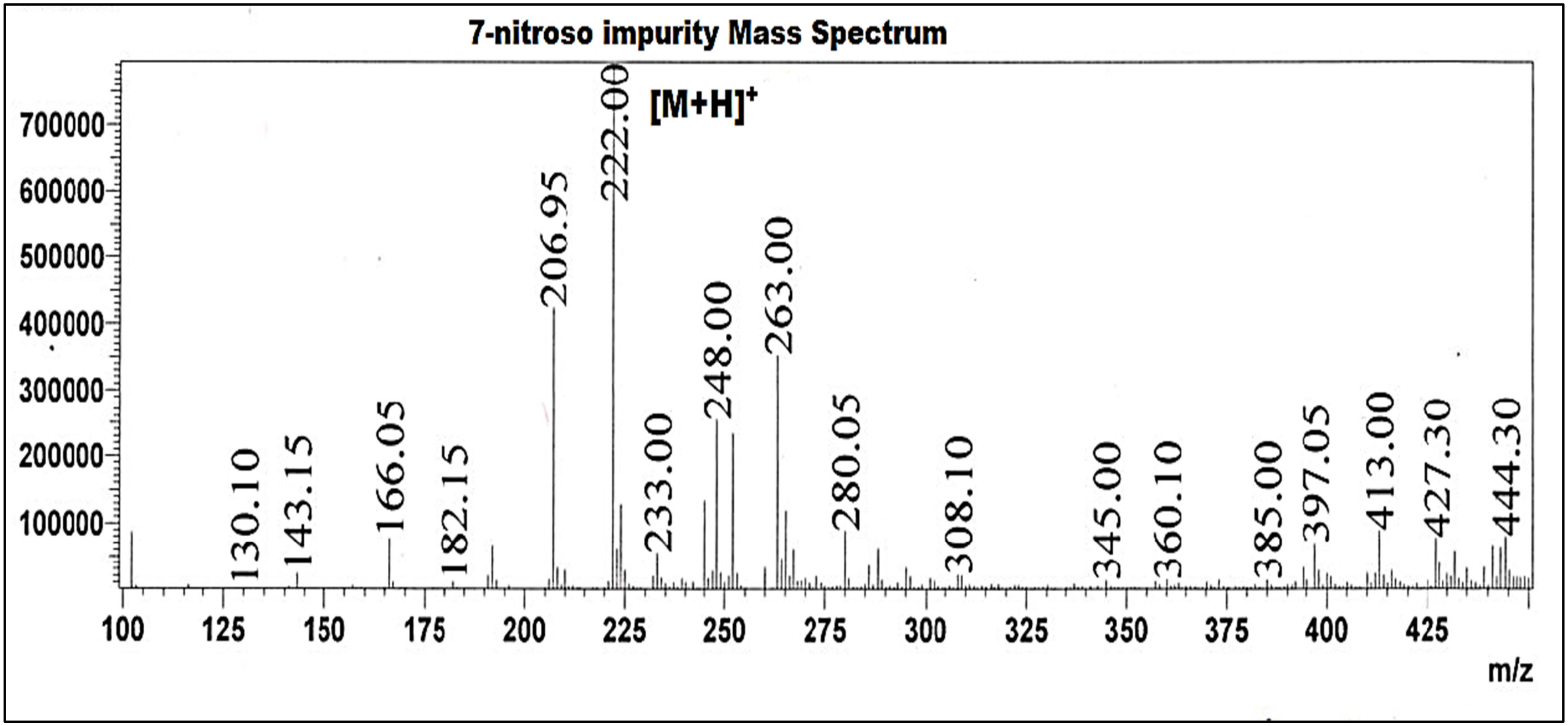

Figure S17: 7-nitroso impurity Mass spectrum.

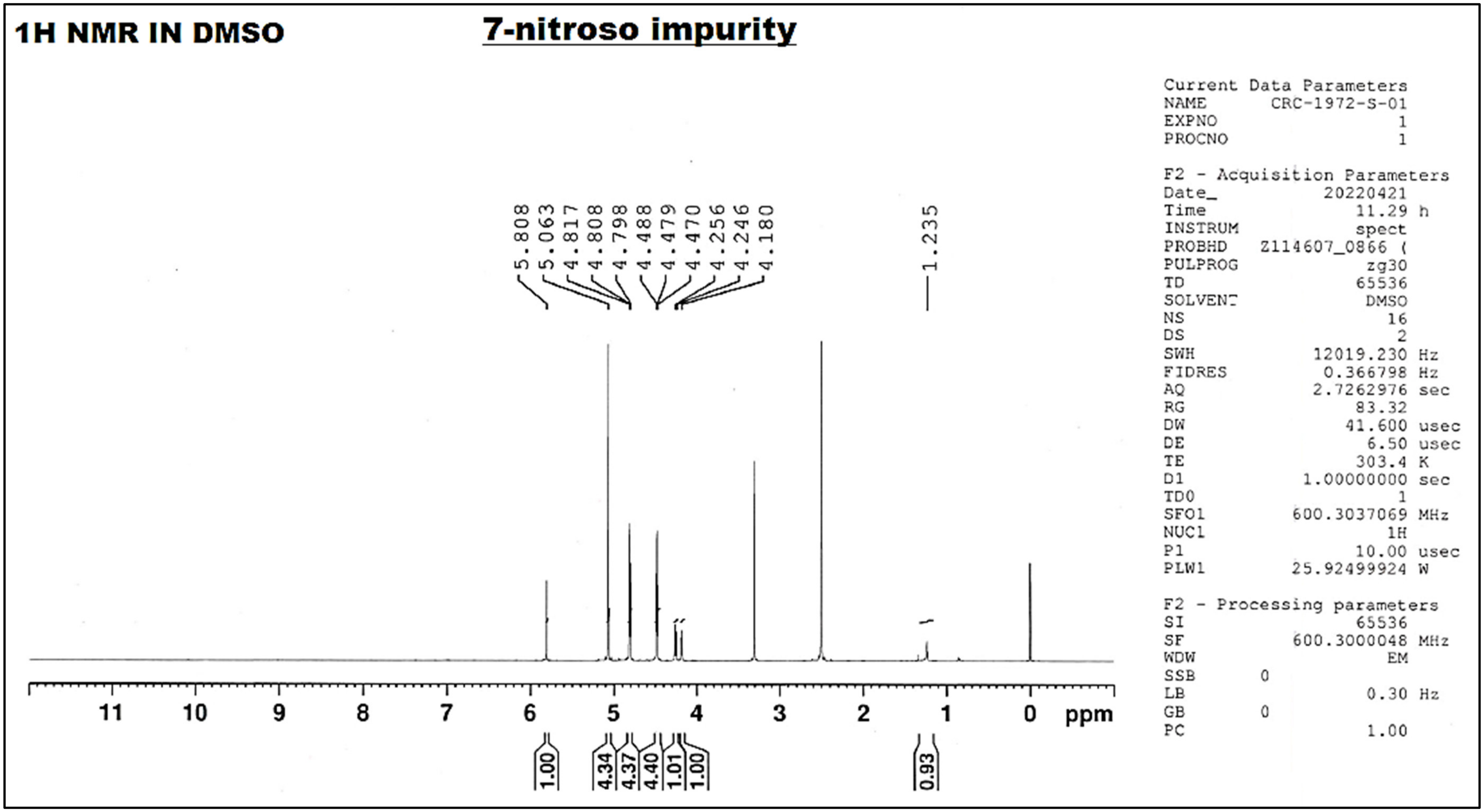

Figure S18: 7-nitroso impurity 1H NMR spectrum – 1.

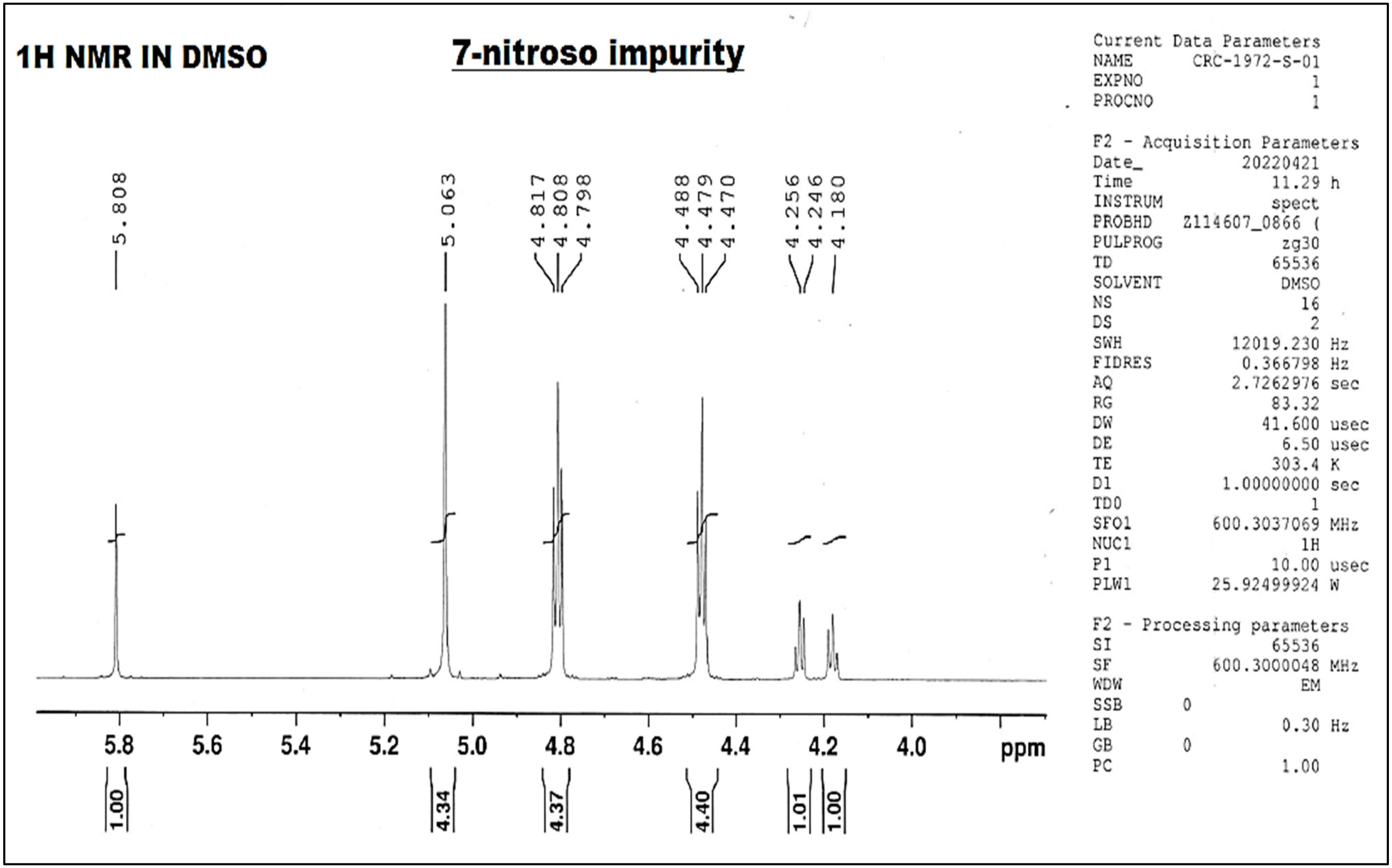

Figure S19: 7-nitroso impurity 1H NMR spectrum – 2.

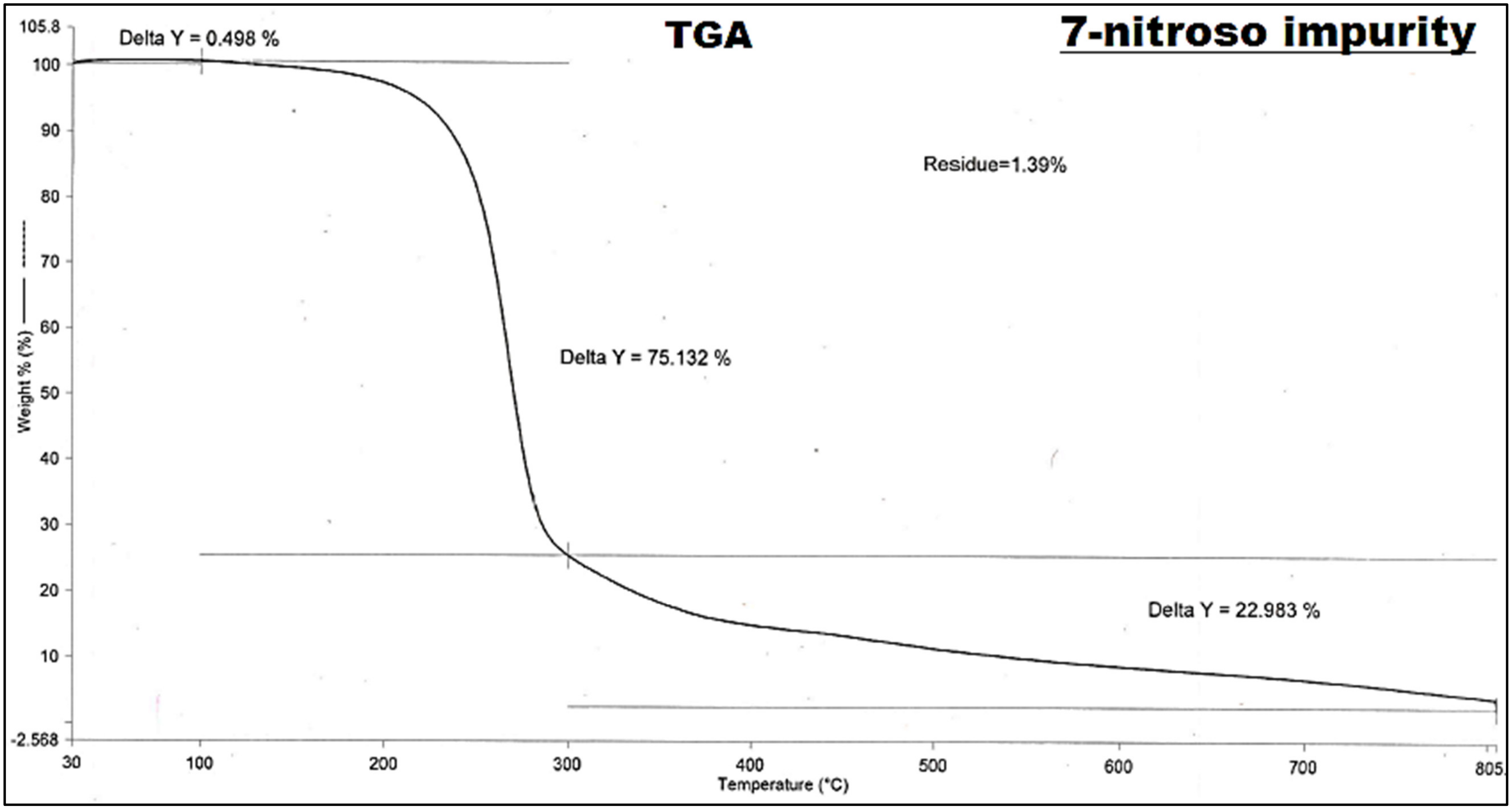

Figure S20: 7-nitroso impurity TGA graph.

7-nitroso-3-(trifluoromethyl)-5,6,7,8-tetrahydro-[1,2,4] triazolo[4,3-a] pyrazine probable parsing pyrolysis fragmentation.

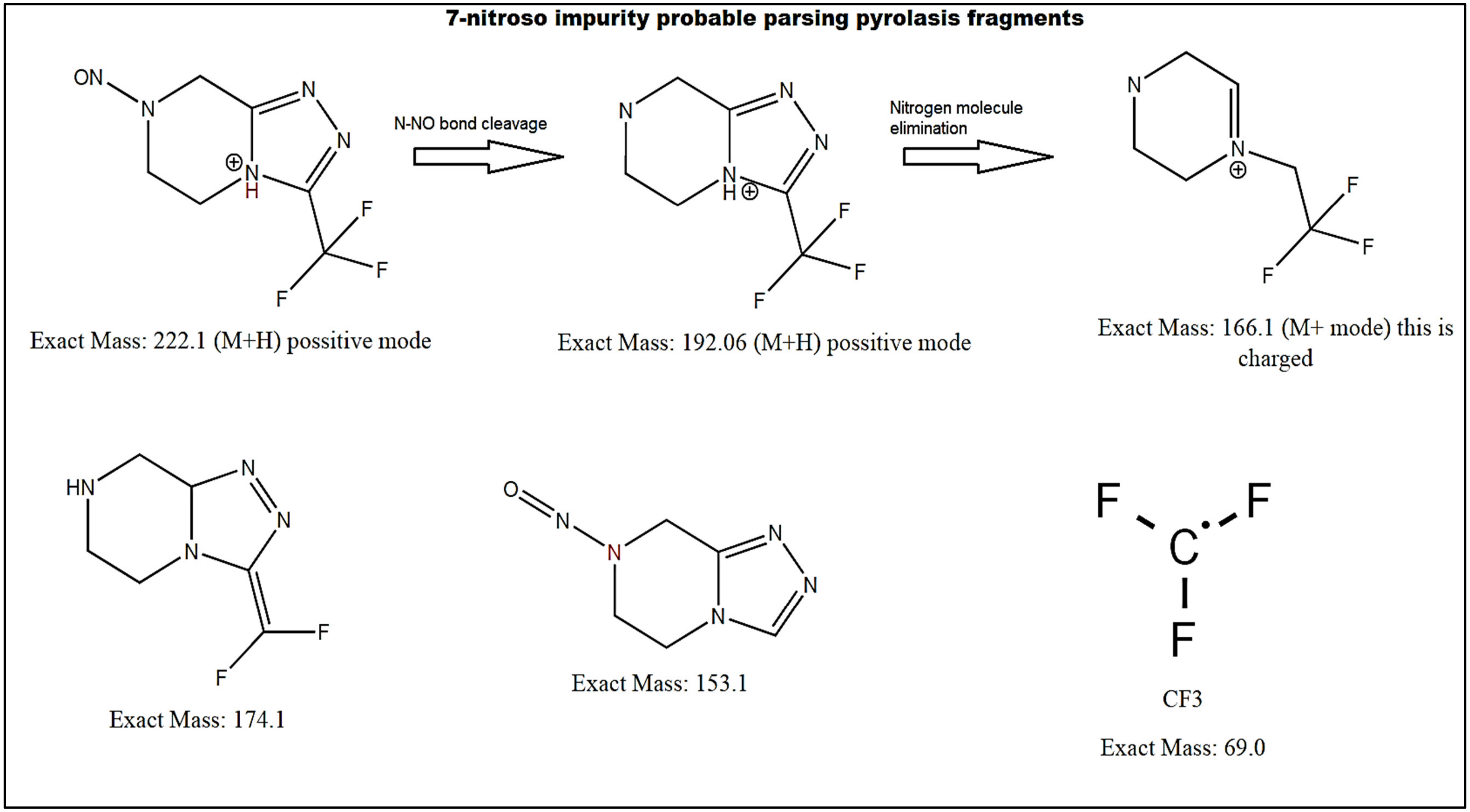

Figure S21: Probable Fragmentation pattern of 7-nitroso impurity in the mass ionization chamber due to parsing pyrolysis.
